# Impact of Printed Circuit Board Dielectric Material on the Thermal Behavior of Wafer-Level Packaging GaN Transistors Used in High-Power-Density Converters for Electric Vehicle Applications

**DOI:** 10.3390/mi17080974

**Published:** 2026-08-18

**Authors:** Mohamed Belguith, Sonia Eloued, Moncef Kadi, Jaleleddine Ben Hadj Slama, Mahmoud Hamouda

**Affiliations:** 1 Laboratory of Advanced Technology and Intelligent Systems, Ecole Nationale d’Ingénieurs de Sousse, Université de Sousse, Sousse 4023, Tunisia; 2 Institut de Recherche en Systèmes Electroniques Embarqués, France Université Rouen Normandie, 76000 Rouen, France

**Keywords:** GaN, dielectric, analytical, Finite Elements Method, thermal, heat dissipation

## Abstract

GaN power devices used in high-power-density converters face significant thermal-management challenges because substantial heat is generated within a compact active region and transferred through the device–Printed Circuit Board (PCB) interface. This study investigates the influence of PCB dielectric-material selection on the coupled thermal and electrical behavior of a 48 V/12 V GaN half-bridge converter. Flame Retardant 4 (FR4), Hydrocarbon ceramic laminate material RO4000 series (4003) (RO4003), and polybenzoxazole (PBO) were compared using a reduced steady-state thermal-resistance network, three-dimensional finite-element simulations in Ansys Icepak, parasitic-capacitance extraction in Ansys Q3D, and switching simulations in LTspice. Both analytical and numerical models produced the same thermal-performance ranking, with PBO providing the lowest junction temperature. Under identical geometry, power dissipation, and boundary conditions, the Finite Elements Method (FEM) results showed a reduction in the maximum junction temperature from 225 °C for FR4 to 159 °C for PBO. The extracted layout-associated capacitances were also reduced by approximately 30–38% with PBO relative to FR4. This decrease produced a slight reduction in the switching-node falling time and lowered the calculated transistor loss from 3.118 W to 3.102 W. The results show that PCB dielectric selection is primarily a thermal-design parameter, while its electrical influence remains modest under the investigated operating conditions.

## 1. Introduction

GaN devices have significant thermal concerns as they are designed to switch high current in a compact package [1]. Due to the high current density and the lateral structure’s tiny conductive channel, the heat dissipation of lateral GaN dies requires careful management. The majority of the heat is produced via the conductive channel of its two-dimensional (2-D) electron gas layer. Even though the substrate is the primary heat-dissipation pathway, a significant portion of the heat remains capable of escaping through the top surface because the 2-D electron gas is in proximity to the electrical pad. Enhanced power module architectures for improving GaN device performance have been the focus of several research studies [2,3,4,5]. GaN HEMTs can be successfully embedded inside the PCB thanks to advancements in PCB manufacturing expertise [6,7,8]. However, this will limit possible cooling topologies. Compared with hybrid strategies, each of the references [9,10,11,12,13,14] suggested a power module design that uses the hybrid PCB-on-DBC structure while including an extra heat-dissipation path to enable a double-sided cooling effect and lower thermal impedance. The top of the design features a multilayer PCB, while the bottom contains a DBC. The advantages of both the highly thermally conductive DBC substrate and the inexpensive PCB are adopted by the hybrid design. In the intermediate layer, two GaN dies are mounted between the DBC on the bottom and the PCB on top. Even though the design’s overall performance is good, it is still not ideal due to its high manufacturing cost and higher weight compared to traditional designs, which makes it unsuitable for deployment in electric vehicle applications. Reference [15] investigated and evaluated several heat-dissipation solutions that have been carried out for GaN-based power converters exhibiting high power density. A detailed analysis was performed on two dissipation systems: a top-side dissipation using various thermal interface materials and a bottom-side dissipation using thermal vias. It is important to note that the transistors possess non-uniform internal temperatures [16,17]. Nonetheless, the junction temperature was decided and kept uniform throughout the device’s interior in order to simplify the model. A similar thermal model was used to predict the thermal behavior of GaN e-HEMTs. Such a thermal model is used to represent the several internal layers or the physical structure of the GaN [18,19].

Compact thermal models are widely used to estimate the junction temperature of power semiconductor devices with lower computational cost than detailed three-dimensional simulations. Górecki et al. reviewed the principal compact thermal-network structures and discussed their application to steady-state and transient thermal analyses of semiconductor devices and power modules [20]. Posobkiewicz et al. showed that the thermal parameters represented by such models depend strongly on mounting conditions, cooling-system properties, and heat-transfer boundaries [21]. Janicki et al. further demonstrated that compact networks can represent both self-heating and thermal coupling among multiple power devices, provided that the relevant heat-flow paths are included [22]. For converter applications, Bahman et al., including Iannuzzo, developed a three-dimensional RC lumped thermal network for high-power IGBT modules that accounts for thermal coupling between chips, package layers, and cooling boundaries while enabling long-term load-profile simulations [23]. These studies confirm the usefulness of reduced thermal networks while emphasizing that their assumptions, boundary conditions, and applicability to steady-state or transient operation must be clearly defined. Recent advancements in GaN gate driver architectures have targeted high-temperature operation, where the integration of deadtime generators enhances switching reliability and thermal robustness [24].

Typically, power converters employ Through Hole Technology (THT) transistors, with relatively long pad connections to the board. Assuming that the thermal flux is unidirectional, a minor proportion of heat flows to the PCB (junction to board) when a heatsink is employed (from junction to heatsink). Due to the pads’ nearness to the board, Wafer-Level Packaging (WLP) GaN transistors, such as the EPC2206 GaN FETs under examination, require the “junction to board” thermal behavior to be taken into account. The temperature of the board is elevated even in the presence of a heatsink. As a result, the heat flux is bidirectional. A significant amount of heat goes into the board despite the limited contact area between the transistors and the heatsink.

One of the common solutions to enhance thermal dissipation is using thermal vias, but they are not a performant solution for high-switching-frequency-based converters since parasitic inductance will increase, which will result in high EMC issues. A lot of studies were performed on high-speed designs (patch antennas, etc.), while less attention was paid to high-power-density designs. Reference [25] investigated the influence of metallization geometry on the electrothermal behavior of power HEMTs using three-dimensional device simulations, showing that structural optimization can significantly affect temperature distribution and device reliability. Reference [26] focused on optimizing the board’s thermal and electromagnetic performance using commercial discrete power GaN FETs (GS61004B). This study was carried out where convection through natural air was strictly forbidden. As a result, adjusting copper track width led to thermal improvement when examining the transistor. Nonetheless, changing the copper track width is limited by dimensions, as a minimum size of the design is required for high-power-density designs, and copper traces are highly thermally conductive and could impact the other components placed next to the transistor.

Reference [27] reviewed PCB design techniques based on increased copper thickness and optimized trace geometry, which can improve heat spreading while contributing to the reduction in electromagnetic emissions in high-frequency GaN converters. In [28], the authors examined three distinct thermal management approaches for GaN HEMTs mounted on PCB substrates. The proposed solutions were Peltier modules, thicker copper traces, and thermal vias. Nevertheless, combining the Peltier module with the high-top-layer copper thickness seemed to be the optimum solution. Using TIMs (thermal interface materials) with excellent thermal conductivity is crucial for high-power-density converters to remove heat from GaN e-HEMT transistors, which are highly compact [15]. In reference [29], the authors studied the impact of THIN LAM® dielectric material on PCB component temperature for high-speed PCB design. Investigations were performed to assess the impact of Thin Lam®, a novel high-thermal-conductivity PCB dielectric material, on the temperature of a PCB voltage regulator MOSFET. As a result, the thermal resistance of the PCB components was reduced by 17% compared to those mounted on the PCB using FR4 dielectric material. In [30], the evaluation of the application of a ceramic substrate with GaN transistors (EPC 2015) demonstrated that ceramic substrates are capable of providing effective thermal management in addition to an appropriate electrical performance (parasitic inductances of 1–2 nH). On the one hand, ceramic substrates are generally heavier and bulkier than conventional PCB dielectric materials. On the other hand, their higher relative permittivity may increase layout-associated parasitic capacitances, which can adversely affect the high-frequency switching behavior and electromagnetic compatibility of the converter. Furthermore, it should be noted that ceramics have a higher thermal conductivity, which will have an impact on the gate driver, which is always placed close to the transistor and the other passive components [31].

In this context, modifying the PCB dielectric material represents a potential approach for improving the thermal management of high-power-density GaN converters. The primary objective of this study is to quantify the influence of dielectric-material selection on the transistor junction temperature using analytical and numerical thermal models. Since the dielectric permittivity also affects the layout-associated parasitic capacitances, these capacitances are extracted and introduced into an electrical switching model to evaluate their influence on the switching-node transition and transistor power losses. The electrical analysis is therefore included to determine whether dielectric-material selection modifies the heat generated by the power devices, thereby complementing the thermal investigation. Conducted and radiated electromagnetic emissions are neither simulated nor measured, and EMI analysis or optimization is outside the scope of the present work.

## 2. Case Study

**Figure 1:**
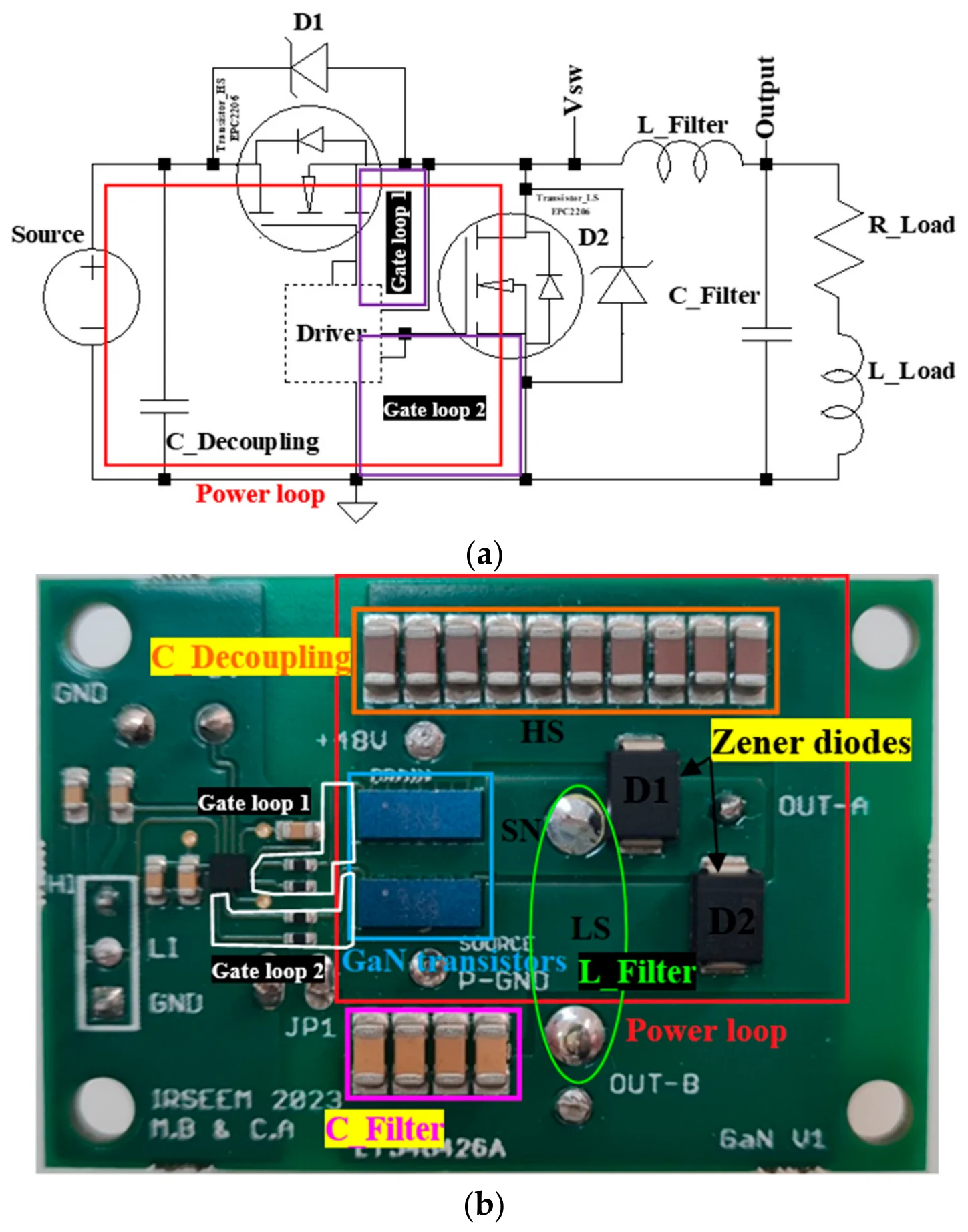
A high-power-density one-sided GaN-based buck converter: (**a**) schematic; (**b**) board.

This research focuses on a case study of a 48 V/12 V-10 A half-bridge buck converter designed using EPC2206 GaN FETs [32], which is designed for application in electric vehicles. The converter has compact dimensions of 6 mm × 2.3 mm and can withstand high currents up to 90 A continuously. It is crucial to study the electrical circuit and depict circuit loops, as shown in Figure 1a, in order to optimize the PCB layout when using high-frequency GaN transistors before the realization of the physical prototype presented in Figure 1b. In order to achieve the desired aim, it is also necessary to study the thermal concerns regarding these transistors.

Wafer-level GaN transistors present a specific thermal-management challenge [33] because the active region is located close to the PCB-facing interconnections and generates heat within a very small area. In contrast to conventional leaded power packages, a significant portion of the heat generated in the device can therefore be transferred toward the PCB through the solder interconnections and copper conductors. In the investigated one-sided converter, the board-directed thermal path can be represented by the successive transfer of heat from the transistor junction through the internal device layers, solder contacts, copper traces, PCB dielectric, and finally the surrounding environment. For an unchanged converter geometry and identical dissipated power, increasing the thermal conductivity of the PCB dielectric is therefore expected to reduce its contribution to the total thermal resistance and consequently decrease the junction temperature.

Nevertheless, replacing the PCB dielectric material is not exclusively a thermal modification. Its relative permittivity affects the electric-field distribution and the layout-associated parasitic capacitances of the power-loop conductors. In high-frequency GaN converters, variations in these capacitances may modify the switching-node transitions and the associated switching losses. Because these losses contribute directly to the heat generated within the transistors, the dielectric materials are evaluated through complementary thermal and electrical analyses. The electrical part of the study is restricted to parasitic-capacitance extraction, switching-waveform analysis, and transistor-loss calculation; it does not constitute an EMI analysis.

To achieve a performant design, it is essential to minimize the gate loop inductance while controlling the GaN HEMTs. Furthermore, the presence of highly parasitic elements in the power loop will result in elevated voltage overshoot and switching losses across the lateral GaN HEMTs, which will reduce their operational lifespan and reliability. However, increasing the thermal conductivity of the PCB dielectric may redistribute heat toward neighboring components, including the gate driver, decoupling capacitors, and control circuitry. This enhanced heat spreading can reduce the local hotspot beneath the GaN transistors, but it may also increase the temperature of nearby temperature-sensitive components, depending on their position, the copper geometry, the PCB thickness, and the available heat-transfer paths. Therefore, the thermal influence of dielectric-material selection on neighboring components should also be considered during PCB design.

**Figure 2:**
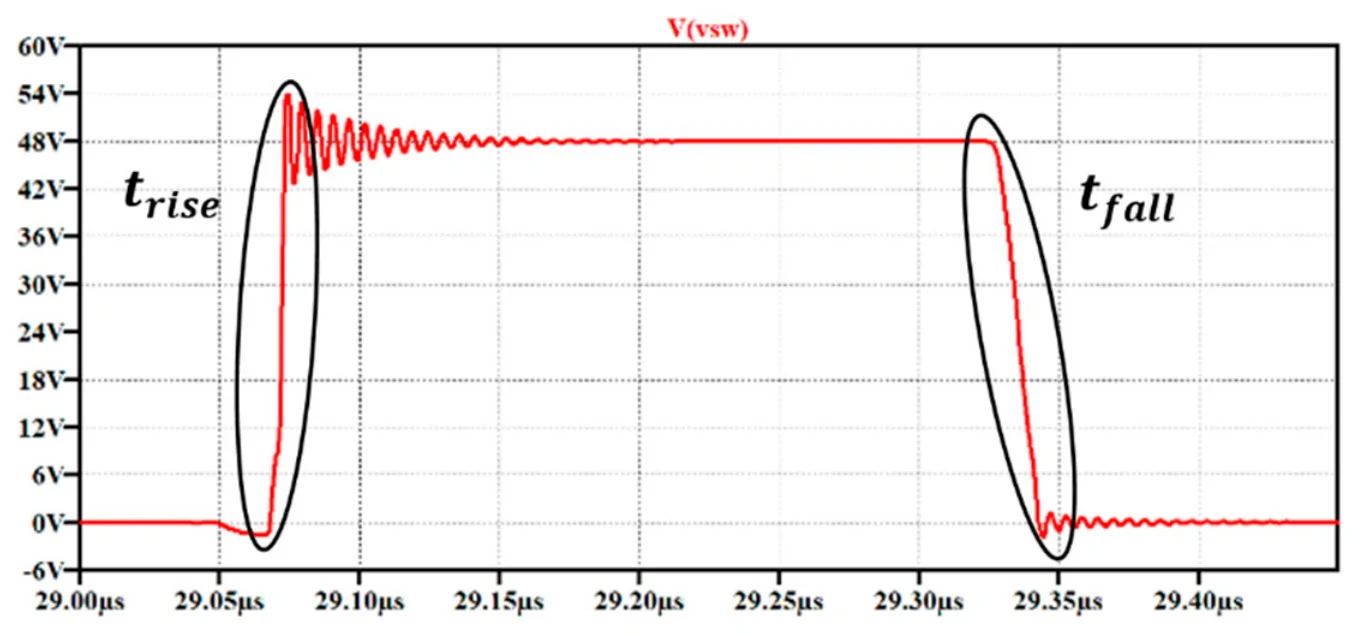
Simulation result of Vsw signal using LTSpice.

In high-frequency GaN converters, the rapid rising and falling transitions of the switching-node voltage Vsw influence the voltage–current overlap during switching and therefore contribute to the transistor switching losses. Figure 2 presents the simulated Vsw waveform used to examine the influence of dielectric-dependent parasitic capacitances on the switching transition.

When a power converter switches off, the energy stored in the circuit’s parasitic inductance causes a voltage spike due to the sudden change in current. This spike can prolong the fall time of the switching signal due to the time required for the energy stored in the inductance to dissipate. Similarly, the parasitic resistance in the circuit can cause voltage drops and slower dissipation of energy, which further contribute to the extended falling time. When a power converter turns off, the parasitic capacitance within the circuitry stores energy. This stored energy can slow down the voltage drop across the circuit during the falling transition of the switching signal.

In high-frequency switching applications or circuits with particularly large parasitic capacitance, the effect of parasitic capacitance on falling time can become more pronounced. The dielectric constant (also known as relative permittivity) of the material affects the capacitance between conductive traces on the PCB. Higher dielectric constants lead to higher capacitance, while lower dielectric constants result in lower capacitance. The distribution of electromagnetic fields between traces is influenced by the dielectric materials used. This has an impact on the mutual inductance between traces, which can affect the overall inductance of the circuit. Furthermore, the thickness and type of dielectric material can impact the spacing between traces, which in turn affects the inductance.

In hard-switched GaN converters, switching losses encompass turn-on/turn-off voltage–current (VI) overlap losses, as well as Eoss (energy stored in output capacitance) and Eqoss (equivalent energy stored in output capacitance) losses. It should be noted that voltage and current overlap losses are specific to hard-switching and are defined by the area where the voltage and current waveforms overlap. These losses occur during both the transistor’s turn-on and turn-off phases. The drain-source capacitances Coss in GaN e-HEMTs result in Eoss and Eqoss losses, which occur exclusively during the turn-on phase [34,35]. Eoss arises from the transistor’s Coss capacitance. Once the transistor has been activated and is in a closed position, the drain-source voltage will decrease, which will cause the Coss to discharge through the transistor. Gaining an understanding of these losses is beneficial for the design of appropriate cooling solutions for the power transistors, which will ensure that they operate within safe temperature limits.

Finally, the PCB provides an extra parasitic capacitance between the source and drain, which increases Eoss and Eqoss losses. The thermal conductivity of standard PCBs, such as those made from FR4 material, is less than 1 W/mK. To address this issue, some PCBs utilize alternative materials with enhanced thermal properties. For example, alumina (Al_2_O_3_) has been proposed in academic studies [30]. It offers significantly enhanced thermal conductivity (24 to 33 W/mK) in comparison to FR4, although it has a relatively low dielectric strength [36]. The high thermal conductivity of these materials can affect the performance of neighboring components, necessitating the use of thicker PCBs when incorporating them. This will increase the overall cost of the design. In order to ascertain the suitability of FR4 materials for embedded power systems, a study was conducted on their thermal behavior and isolation qualities [37]. An IGBT was implemented, and several PCB materials were assessed. The temperature-dependent thermal conductivity of each material was determined, and experiments were conducted to assess the temperature-dependent partial discharge characteristics.

## 3. Methodology

Our objective is to enhance thermal management, including the use of a heatsink, by employing a better and more performant dielectric material that will optimally absorb heat without impacting the converter’s other components or transistor reliability. The aim of this study is to reduce the thermal resistance associated with the PCB heat-transfer path and, consequently, the cooling effort required to maintain the transistor junction temperature below a prescribed limit. Although the present work does not directly optimize the heatsink geometry, a lower junction temperature obtained with an improved dielectric material increases the allowable thermal resistance of the external cooling system, which may permit the use of a smaller and lighter heatsink under equivalent cooling conditions [38].

**Figure 3:**
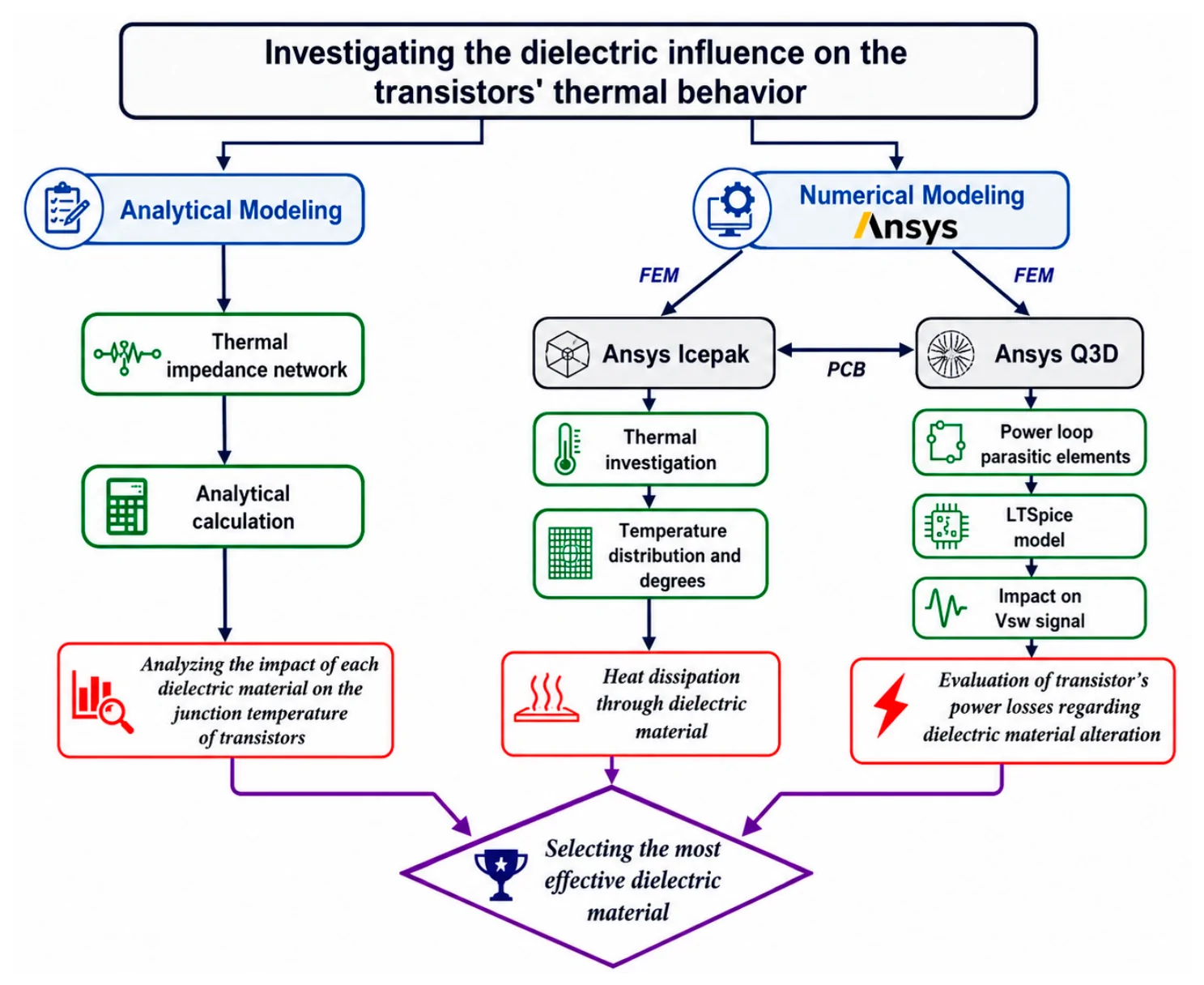
Flowchart of the methodology adopted in this study.

The flowchart shown in Figure 3 summarizes the methodology adopted in this study.

Our approach is to evaluate the impact of three distinct dielectric materials on the thermal behavior of GaN transistors.

The first material is a typical FR4 dielectric. The second material is a hydrocarbon ceramic laminate material “RO4000” series (4003). The third material is a polybenzoxazole (PBO) dielectric material. The structure currently under study is analytically modeled, and extensive details are provided. Additionally, an FEM simulation using Ansys Icepak is generated during this study to evaluate the substances within a model of a GaN-based high-power-density converter [39]. Moreover, Ansys Q3D simulations were performed to extract the power-loop parasitic capacitances associated with each dielectric material. Since these parasitic capacitances influence the switching-node transitions, the extracted values were introduced into the LTspice converter model to evaluate their effect on the Vsw waveform and the resulting transistor switching losses. This electrical analysis complements the thermal investigation by determining whether dielectric-material selection also modifies the heat generated by the GaN devices.

**Figure 4:**
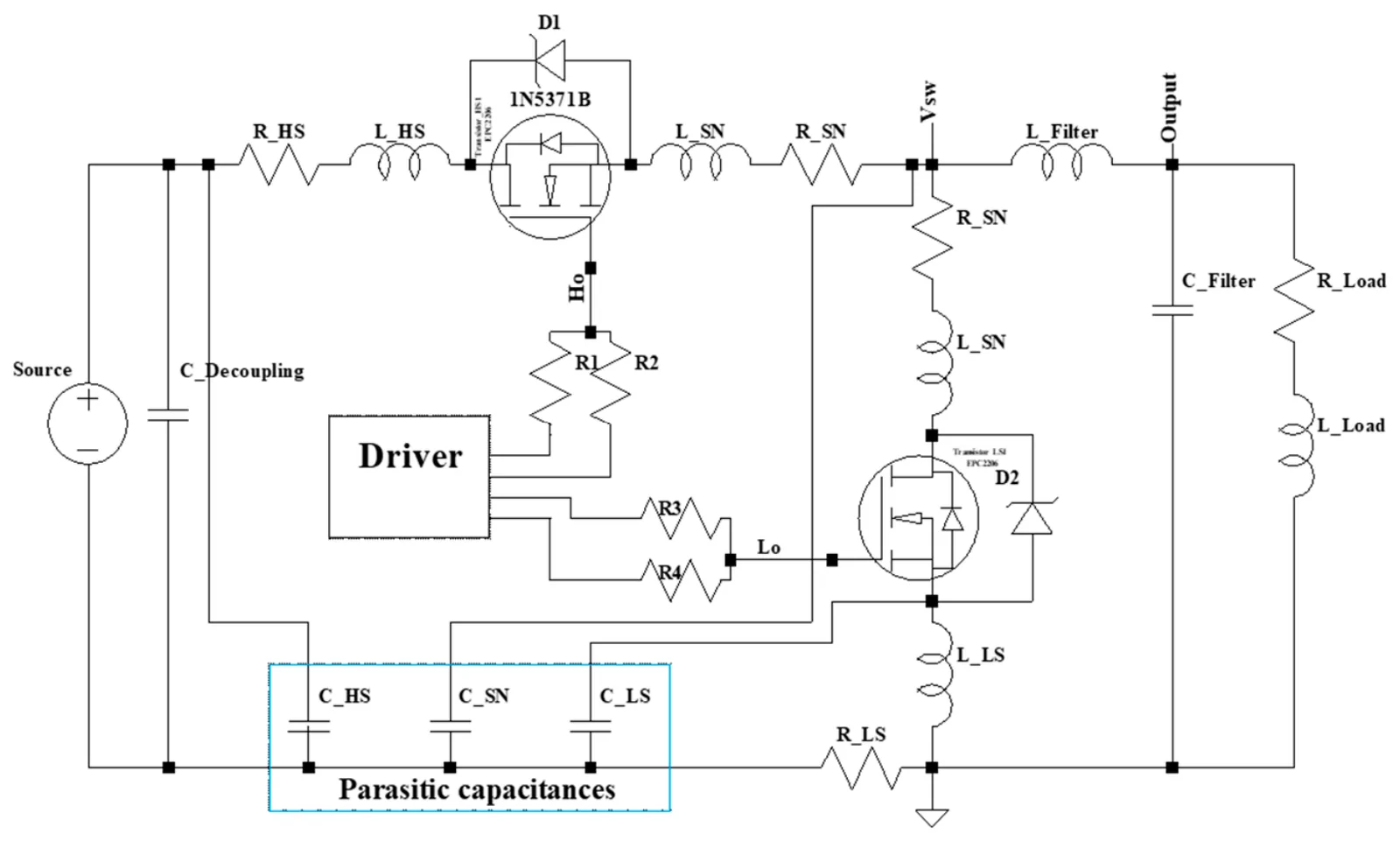
Buck converter model in LTSpice.

FR4 (Flame Retardant 4) is a grade designation for glass-reinforced epoxy laminate material used in the manufacturing of printed circuit boards (PCBs). It is well-known for its excellent mechanical strength, electrical insulation, and thermal properties. The “RO4000” series, which includes “RO4003”, consists of high-frequency laminates tailored for performance-oriented PCB applications. The mechanical characteristics of conventional epoxy/glass laminates are combined with the electrical properties of high-frequency ceramics in “RO4003”. Its high thermal conductivity ensures efficient heat dissipation during use. These laminates offer higher-performance materials at a lower cost than conventional PTFE-based laminates due to their distinctive combination of hydrocarbon and thermoset resins infused with ceramic particles. Polybenzoxazoles (PBOs) are high-performance dielectric materials renowned for their outstanding thermal and mechanical properties. PBOs are ideal for sophisticated electrical and industrial applications due to their specific characteristics. PBOs are ideally suited for applications that require long-term thermal stability and can sustain their performance without degrading at high temperatures since they can endure such temperatures. The relative permittivity values of the three investigated dielectric materials are summarized in Table 1:

The LTSpice simulation model for each dielectric material is illustrated in Figure 4. This includes the command section (driver), power loop, filter section, and power-loop-parasitic elements of the converter, which were extracted using Ansys Q3D. The principal differentiations between the dielectric materials pertain to the parasitic capacitances, which will be incorporated subsequently in the model along with the other parasitic elements in order to ascertain their impact on the Vsw signal and determine the power losses of the transistor for all dielectric material cases.

**Figure 5:**
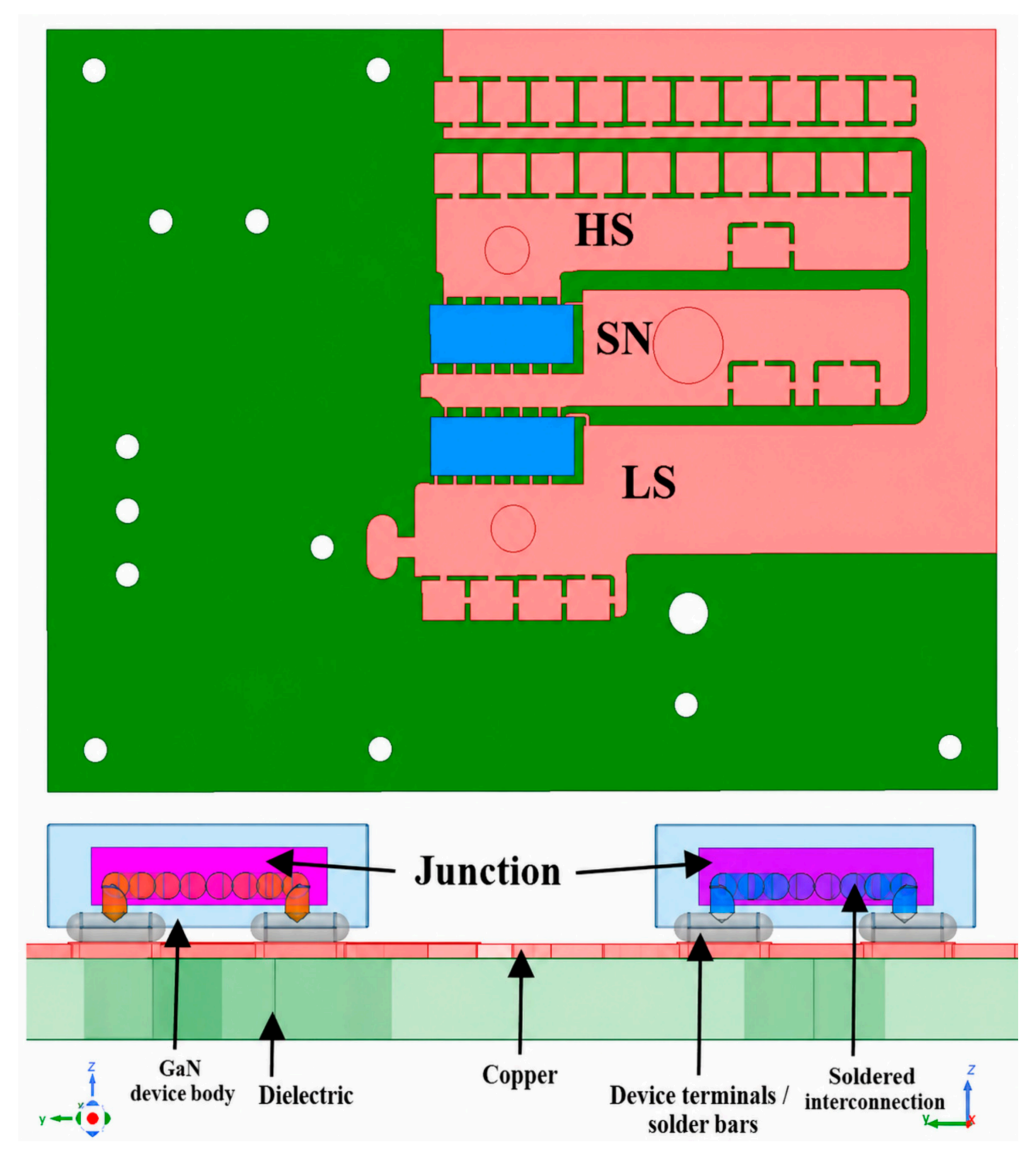
Three-dimensional electrothermal model of the GaN half-bridge converter in Ansys Icepak.

For the thermal simulations performed in Ansys Icepak, a three-dimensional numerical model of the converter was developed, as illustrated in Figure 5. The model includes the power-loop copper conductors, PCB dielectric layer, soldered transistor interconnections, and the two wafer-level GaN devices. The power-loop copper regions were modeled explicitly because they constitute the principal heat-spreading paths between the devices and the PCB.

Each GaN device is represented by an active heat-generating region, an internal device body, and the soldered interconnections linking its electrical terminals to the copper footprint [40]. Heat generated within the active region is transferred toward the PCB through the internal device structure and soldered contacts, while additional heat-transfer paths toward the surrounding environment are accounted for through the numerical boundary conditions. This representation is consistent with the wafer-level mounting configuration and avoids introducing conventional wire bonds or leaded-package elements that are not present in the investigated device.

## 4. Results and Discussion

This section compares the three dielectric materials using analytical thermal modeling, three-dimensional FEM simulations in Ansys Icepak, and transistor-loss calculations based on the Q3D-extracted parasitic capacitances.

### 4.1. Analytical Modeling

Analytical modeling is employed to compare the influence of the three dielectric materials on the thermal behavior of the GaN transistors. For this purpose, the converter structure is transformed into an equivalent thermal network representing the successive heat-transfer paths from the transistor junction to the PCB and the surrounding environment. The model provides a first-order estimation of the junction temperature and establishes a physical basis for comparison with the subsequent FEM simulations.

Heat transfer through the converter is represented using an equivalent thermal network composed of thermal resistances and thermal capacitances. Thermal resistance describes the opposition of a material layer to steady heat conduction, whereas thermal capacitance represents its ability to store thermal energy during temperature variations. These two parameters provide the basic physical quantities required to describe both the steady-state and transient thermal responses of the system.

The thermal resistance of a homogeneous material layer is expressed as

(1) $$R_{th}=\frac{L}{KA}$$

where

- $R_{th}$ is the thermal resistance;
- L is the thickness of the material;
- K is the thermal conductivity;

A is the cross-sectional area (the area through which heat is being transferred).

Thermal capacitance:

(2) $$C_{th}\;=\;\rho Vc\;=\;mc$$

where

- $C_{th}$ is the thermal capacitance;
- *ρ* is the density of the material;
- *V* is the volume; *c* is the specific heat capacity;
- *m* is the mass of the material.

Equation (1) shows that the thermal resistance increases with material thickness and decreases with thermal conductivity and heat-transfer area. Equation (2) shows that the thermal energy-storage capability depends on the mass and specific heat capacity of the material.

Equations (1) and (2) describe the conductive resistance and thermal storage of the solid material layers only. Convection and radiation are not represented by these expressions. In the reduced analytical network, heat exchange between the exposed PCB surface and the ambient environment is represented by an equivalent external thermal-boundary resistance that collectively accounts for convection and radiation. The detailed spatial distribution of these heat-transfer mechanisms and the multidirectional heat-flow paths are represented more accurately in the subsequent three-dimensional FEM model.

In the investigated wafer-level GaN device, heat generated within the active region is transferred toward the PCB through the internal device structure, soldered interconnections, and copper conductors. These solid regions provide the principal board-directed conductive path and are therefore represented by their corresponding thermal resistances and capacitances.

Although both thermal resistance and thermal capacitance are introduced in Equations (1) and (2), the present analytical calculation is restricted to steady-state conditions. Under constant power dissipation, thermal capacitances govern the time required to reach thermal equilibrium but do not determine the final equilibrium temperature. Consequently, Equation (2) is retained to define the complete thermal behavior of the material layers, whereas only the thermal resistances are used in the steady-state junction-temperature calculation. A transient thermal analysis would require both the $R_{th}$ and $C_{th}$ elements.

**Figure 6:**
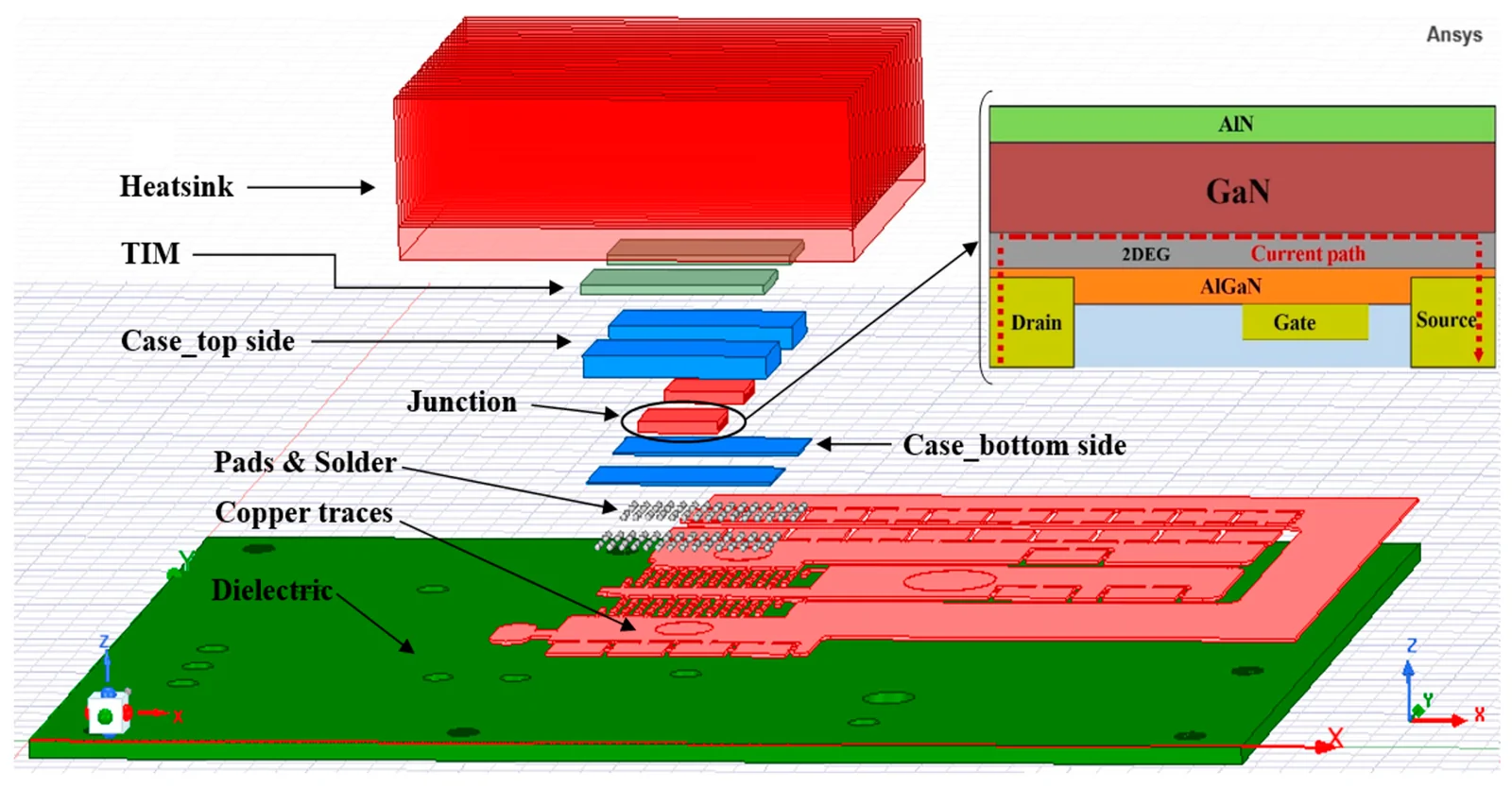
General design structure of the converter including a heatsink.

**Figure 7:**
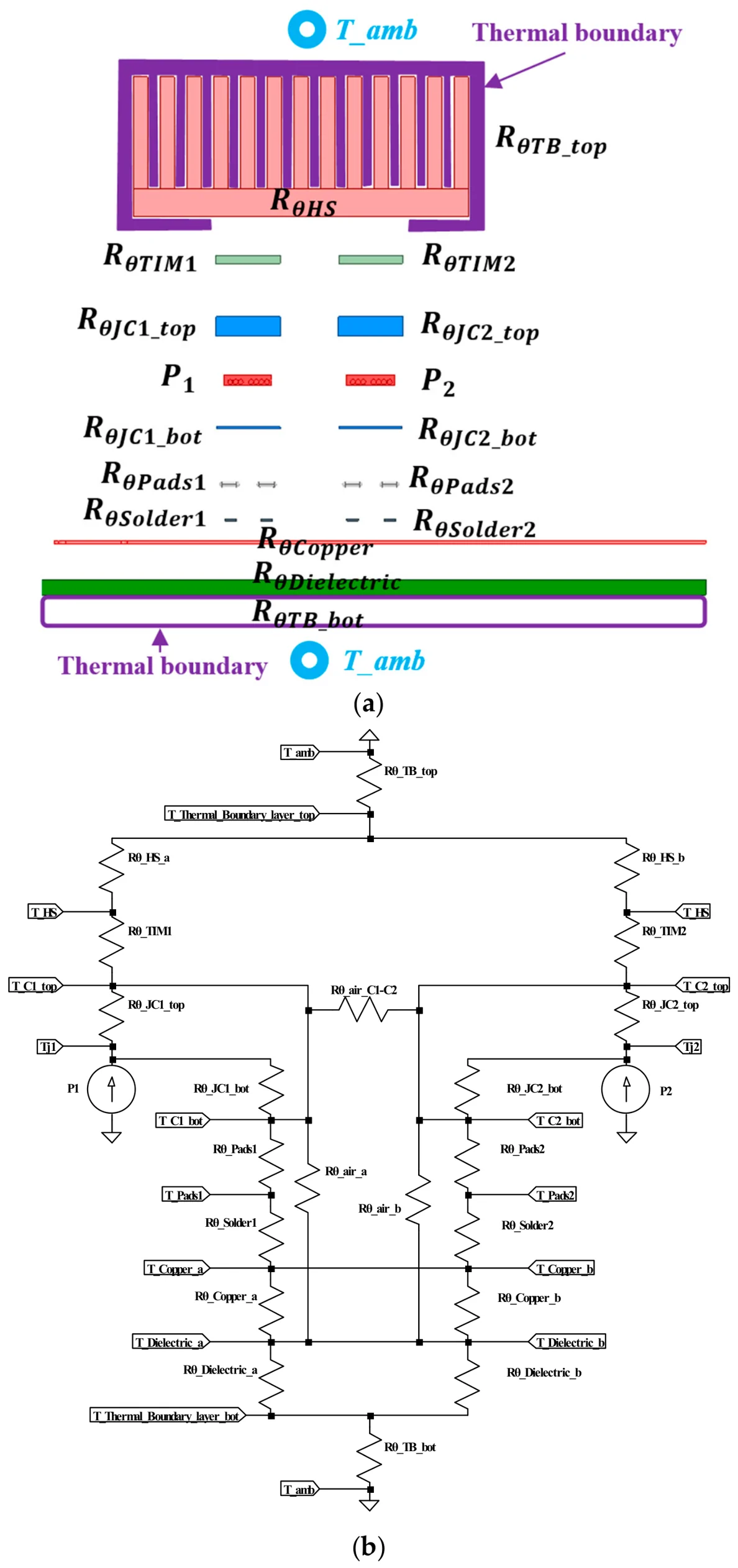
Transformation of the complete converter thermal structure into an equivalent thermal-resistance network: (**a**) physical representation of the main heat-transfer paths through the heatsink, transistor packages, soldered interconnections, copper regions, PCB dielectric, and external thermal boundaries; (**b**) corresponding lumped thermal-resistance network including the two GaN devices, top- and bottom-side heat-transfer paths, inter-device thermal coupling, and ambient thermal boundaries.

The general design structure, including a heatsink and its modeling and transformation into a thermal resistance network, is illustrated in Figure 6 and Figure 7.

The enlarged illustration in Figure 7 demonstrates the transistor’s junction architecture, which encompasses AlN, the GaN layer, and other constituent layers. Given that the AlN layer is relatively thin in comparison to the SiC layer, the substrate in this case will be represented by the SiC layer along with the passivation layer, referred to as “case_top side” [37]. On the other hand, the “case_bottom side” presents the passivation layer on the bottom side, so the surface state trapping effect will not be considered.

$R_{\theta \_air\_C1\_C2\;}$ represents the air thermal convection between the two transistor cases. $R_{\theta \_air\_a\;}$ denotes the thermal air convection between the bottom part of the first transistor case and the dielectric region just under the first transistor (same topology for transistor 2). $R_{\theta \_Copper\_a\;}$ represents the copper region in direct contact with the first transistor pads. This is also where we found the solder (Tin) (same topology for transistor 2). $R_{\theta \_TB\;}$denotes the thermal boundary, or the hot air region in direct interaction with the dielectric.

**Figure 8:**
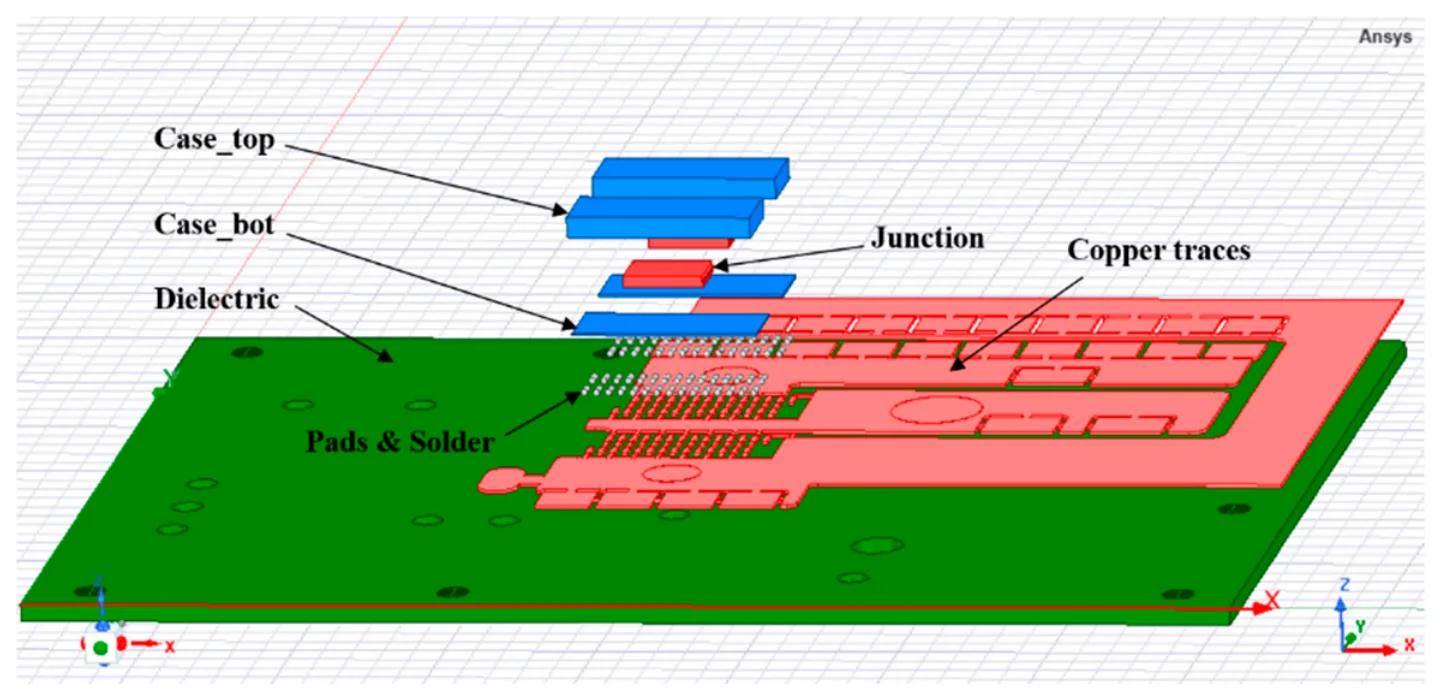
Reduced board-directed heat-transfer structure considered in the analytical model.

To isolate the contribution of the PCB dielectric material, the reduced analytical model considers only the board-directed heat-transfer path. Heat generated in the transistor is therefore represented as passing through the internal device region, soldered interconnections, copper conductors, PCB dielectric, and the lower external thermal boundary, as illustrated in Figure 8.

**Figure 9:**
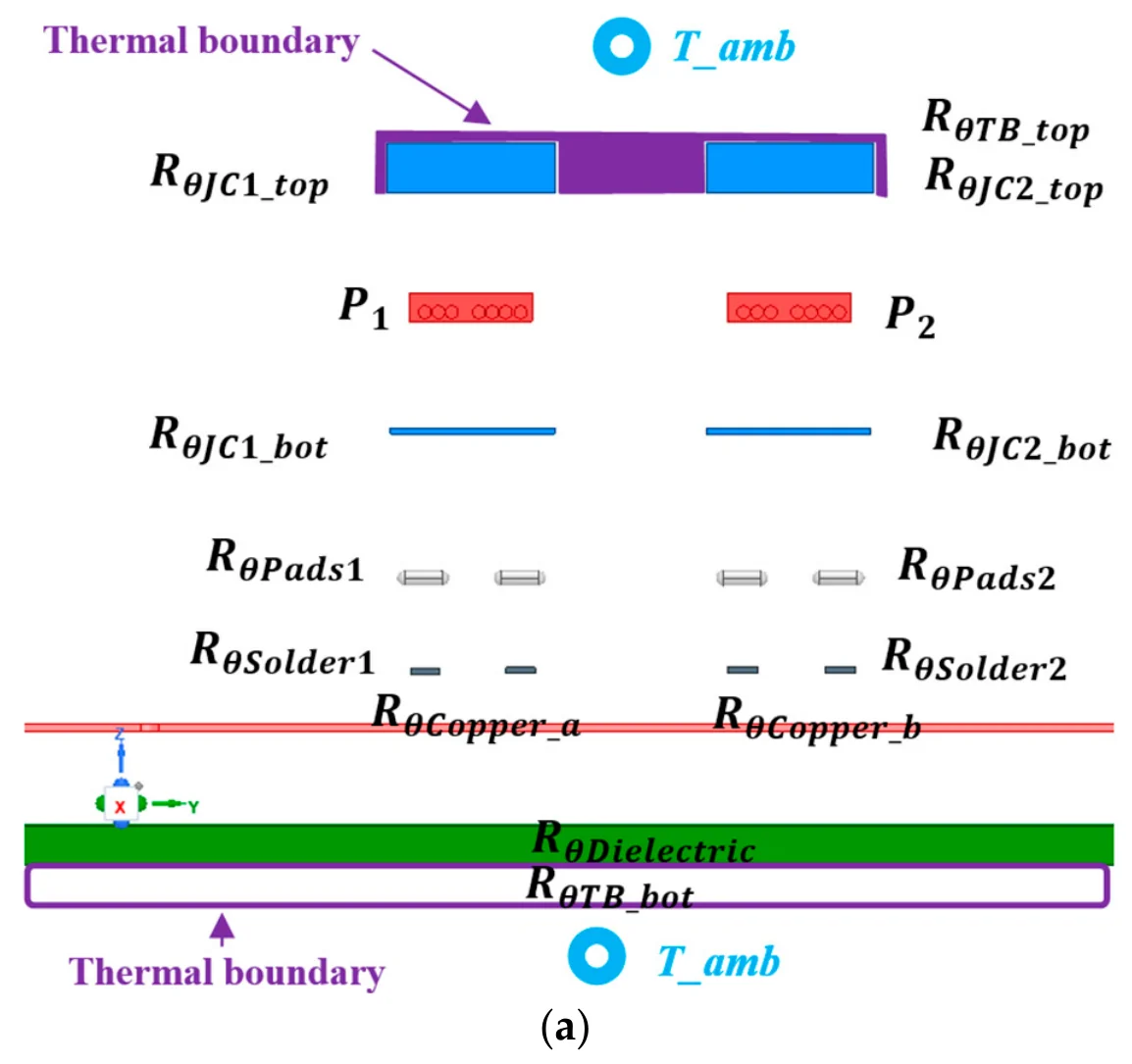

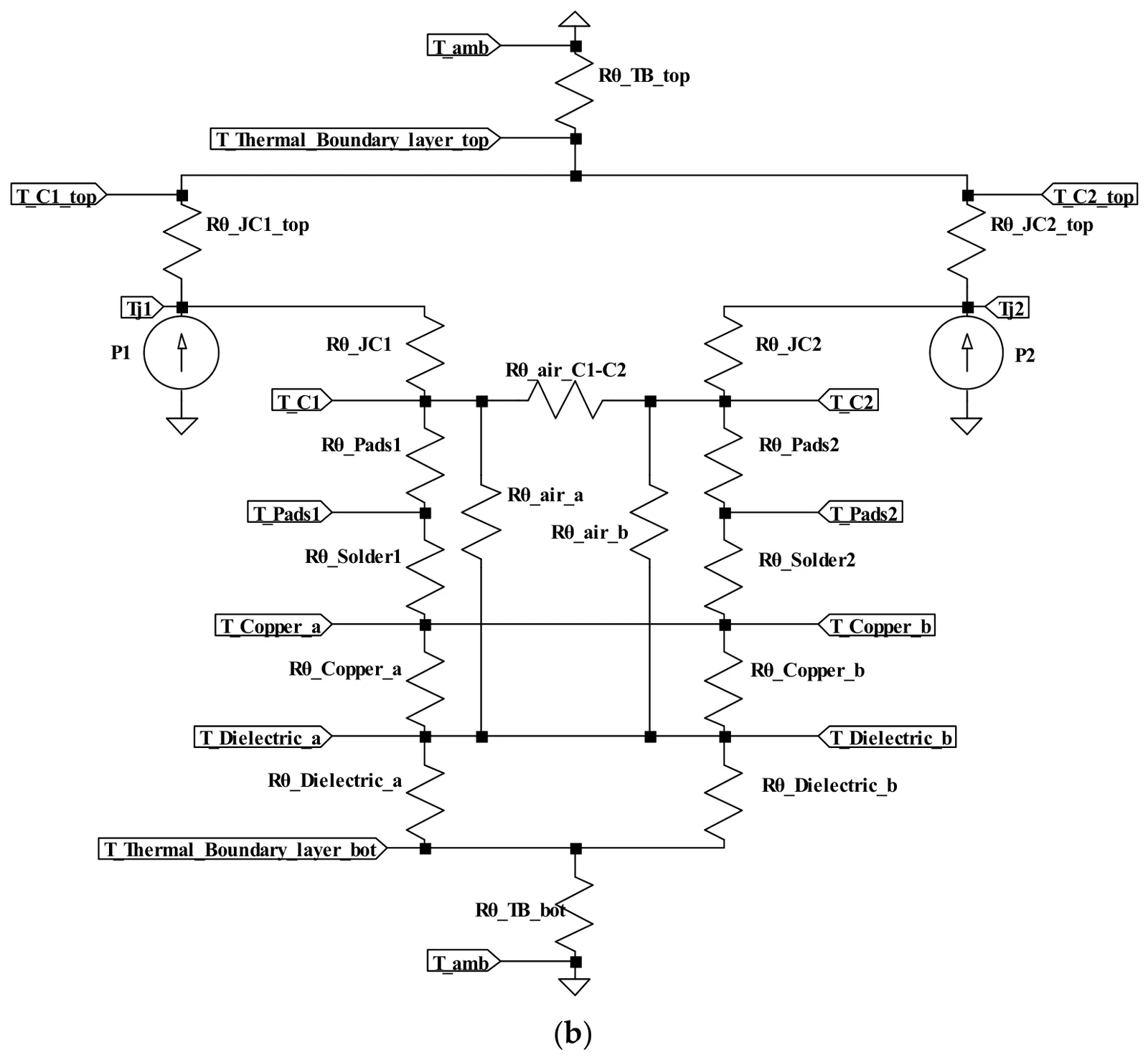
Transformation of the reduced board-directed thermal structure into its equivalent analytical network: (**a**) simplified physical representation retaining the heat-transfer paths from the two transistor junctions through the device cases, soldered contacts, copper regions, PCB dielectric, and lower thermal boundary; (**b**) corresponding equivalent thermal-resistance network including the two device branches, their thermal coupling, and the common lower ambient boundary.

This simplification does not imply that all the heat generated by the transistor is physically transferred toward the PCB. In the complete converter, heat may also be dissipated through the device top surface, neighboring conductors, surrounding air, and any attached cooling structure. These additional paths are omitted from the analytical network so that the influence of the dielectric material can be examined independently. The complete multidirectional heat-flow behavior is subsequently evaluated using the three-dimensional FEM model. The resulting reduced physical representation and its corresponding equivalent board-directed thermal-resistance network are presented in Figure 9.

**Figure 10:**
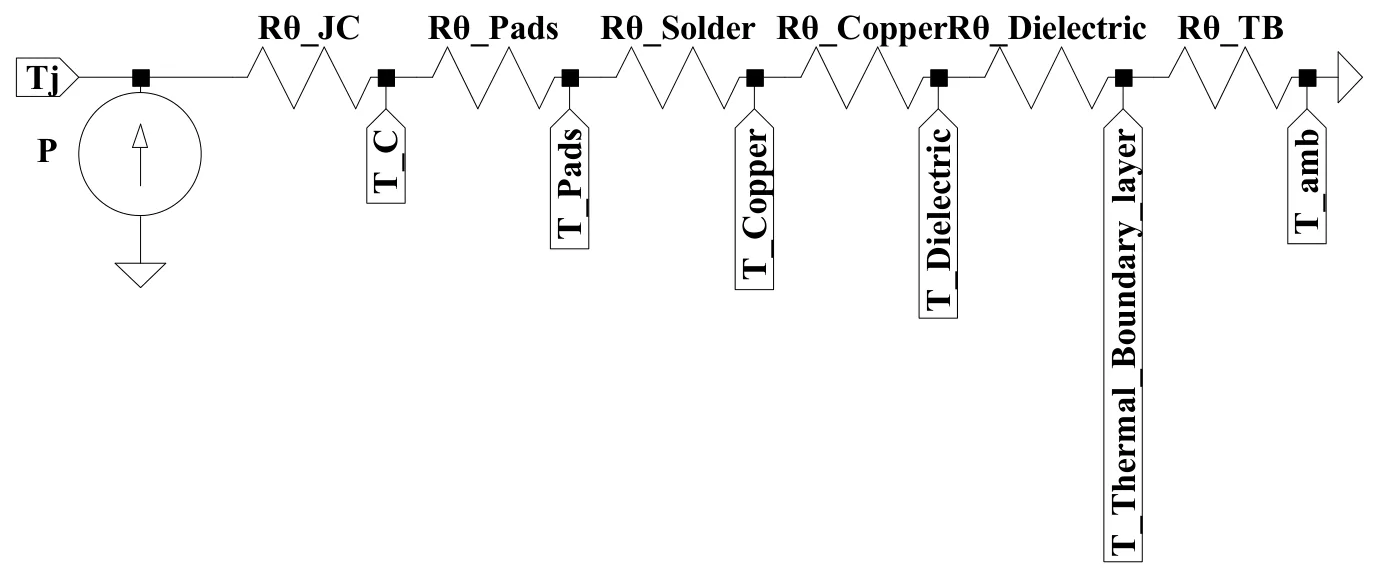
Thermal resistance network of the converter’s model of study.

Since the two GaN transistors have identical geometries and are assigned the same dissipated power and external boundary conditions in the analytical study, their board-directed thermal paths are assumed to be equivalent. The half-bridge can therefore be represented by the reduced single-transistor thermal network shown in Figure 10. This simplification is used only for the comparative analytical model. If the two devices experience different conduction intervals, switching losses, copper environments, or cooling conditions, separate thermal networks must be considered. The equivalent network represents one device only; the total converter thermal response must account for both devices when their dissipated powers or thermal environments are not identical.

The junction temperature will be determined using Equation (3), which takes into account power dissipation and the different levels of thermal resistance $R_{\theta JA}$ calculated in Equation (4).

(3) $$T_{J}=P_{Loss}\;*\;R_{\theta JA}+T_{amb}$$

where

- $\;T_{J}$ is the junction temperature;
- $\;P_{Loss}$ is the total dissipated power;
- $T_{amb}$ is the ambient temperature;
- $R_{\theta JA}$ is the total thermal resistance.

(4) $$R_{\theta JA}=R_{\theta JC}+R_{\theta Pads}+R_{\theta Solder}+R_{\theta Copper}+R_{\theta Dielectric}+R_{\theta \_TB\;}$$

#### 4.1.1. Modeling Assumptions and Physical Basis

The analytical thermal network is developed as a reduced steady-state model for comparing the influence of the three PCB dielectric materials. Its objective is to identify the physical contribution of the dielectric layer to the board-directed heat-transfer path rather than to reproduce the complete three-dimensional temperature distribution of the converter. The following assumptions define the scope and validity of the model.

**Steady-state thermal conditions:** The transistor power dissipation and ambient conditions are assumed to remain constant until thermal equilibrium is reached. Thermal capacitances are therefore neglected because they govern the heating and cooling time constants but do not directly determine the final steady-state temperature. Consequently, the analytical model is intended for equilibrium-temperature comparison and not for transient thermal cycling or short-duration overload prediction.

**Controlled comparison between dielectric materials:** The transistor geometry, solder interconnections, copper layout, dielectric thickness, ambient temperature, dissipated power, and external cooling conditions are kept identical for the FR4, RO4003, and PBO cases. The thermal conductivity of the dielectric is therefore the principal variable affecting the analytical thermal path. This controlled comparison ensures that the predicted temperature differences can be attributed primarily to the dielectric material rather than to variations in geometry or operating conditions.

**Board-directed heat-transfer path:** The analytical model considers the dominant heat-transfer path from the transistor junction toward the PCB. Heat is transferred successively through the internal device region, the soldered interconnections, the copper conductors, and the PCB dielectric, and finally from the exposed PCB surface to the ambient environment. Heat transfer through the transistor top surface is neglected in this reduced model to isolate the contribution of the PCB path. This does not imply that the complete converter transfers heat exclusively toward the board; the additional multidirectional heat-flow paths are considered in the subsequent FEM model.

**Internal device thermal resistance:** The thermal resistance associated with the internal device structure is obtained from the manufacturer data. This resistance represents the heat-transfer path between the active region and the device mounting interface. It is connected to the external PCB thermal network without separately reproducing internal layers already included in the manufacturer-provided value, thereby avoiding double counting of the device thermal resistance.

**Soldered-interconnection model:** Each thermally active interconnection between the transistor and the PCB is represented by an individual conductive thermal resistance. Interconnections connecting approximately identical thermal nodes are combined in parallel. The number and dimensions of these thermal branches are obtained from the implemented transistor footprint and the CAD geometry.

The solder layer is represented using an average thickness of 60 µm. Because the solder distribution beneath each interconnection is not perfectly uniform, an effective contact area equal to one-half of the nominal interconnection area is considered. This geometric approximation is applied identically to all three dielectric cases. It may influence the absolute calculated temperature, but its influence on the relative comparison between the materials is reduced because the solder geometry remains unchanged.

**Copper heat spreading:** Heat transferred through the soldered interconnections enters several copper regions associated with the transistor footprint. The conductive resistance of each copper path is calculated from its thickness, thermal conductivity, and effective cross-sectional area. Copper paths connected to different footprint regions are retained as separate branches, while equivalent paths connecting the same thermal nodes are combined in parallel. The analytical network neglects detailed lateral heat spreading and local temperature gradients within the copper; these effects are represented more accurately in the three-dimensional FEM model.

**Dielectric thermal resistance:** The dielectric layer is modeled using the dielectric thickness, its thermal conductivity, and its effective area through which heat is transferred from the copper to the PCB underside. The dielectric thickness and effective heat-transfer area are maintained constant for all material cases. Therefore, the change in dielectric thermal resistance is directly governed by the thermal conductivity of FR4, RO4003, or PBO.

**External thermal boundary:** Heat transfer from the lower PCB surface to the surrounding environment is represented in the reduced analytical model by an equivalent external thermal-boundary resistance. This lumped resistance accounts collectively for the heat-transfer mechanisms occurring between the heated PCB region and the ambient environment. The same external-boundary resistance is applied to the FR4, RO4003, and PBO configurations so that the comparison isolates the influence of the dielectric material.

Because this equivalent resistance does not reproduce the complete local convection and radiation phenomena, the analytical temperatures are interpreted primarily as comparative estimates. The detailed external boundary conditions and multidirectional heat-transfer paths are represented more accurately in the subsequent three-dimensional FEM model.

**Temperature-independent material properties:** The thermal conductivities of the solid materials are initially assumed to remain constant over the investigated temperature range. This approximation simplifies the analytical comparison. The effect of temperature-dependent material properties and three-dimensional heat spreading is subsequently evaluated through the FEM model.

**Figure 11:**
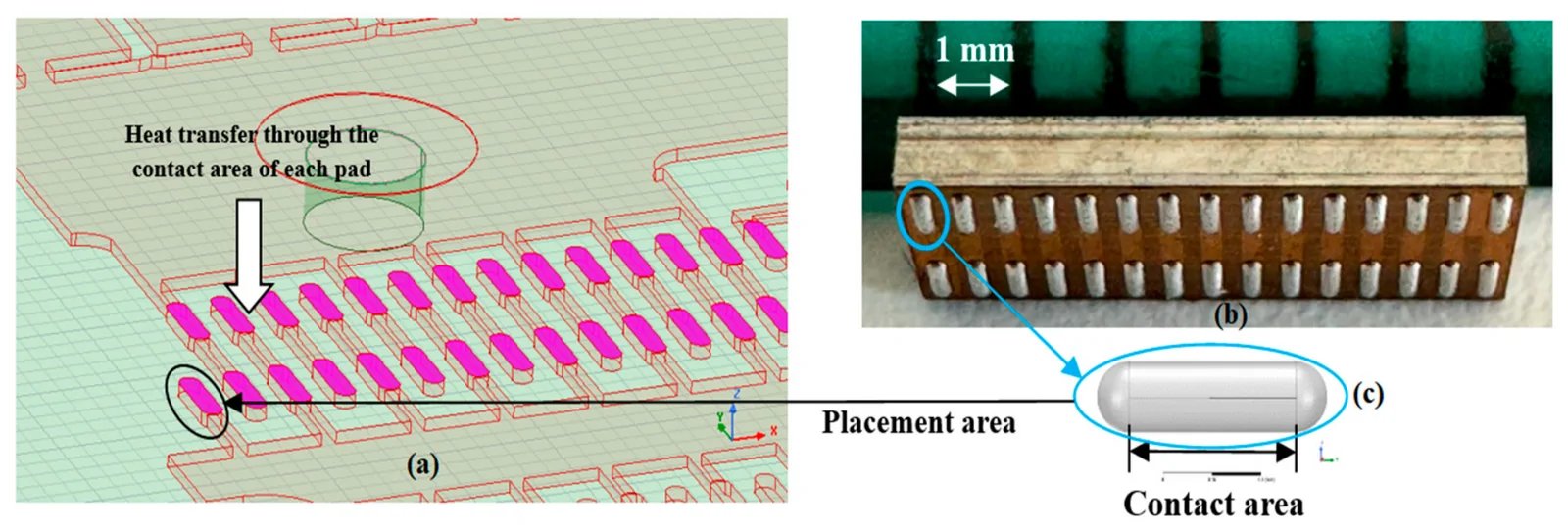
Transistor’s contact and mounting technique on the PCB: (**a**) EPC2206 footprint in copper tracks; (**b**) EPC2206 GaN transistor; (**c**) transistor pad.

Based on these assumptions, the analytical model is primarily used to compare the relative thermal performance of the dielectric materials. The FEM results are then employed to evaluate the complete temperature distribution and verify whether the material ranking predicted by the reduced thermal network remains valid. The EPC2206 footprint, transistor package, and effective contact area used to define the transistor-to-PCB thermal paths are illustrated in Figure 11.

#### 4.1.2. Calculation

The thermal resistance of each layer and interconnection region was calculated using Equation (1) and the geometrical dimensions extracted from the converter CAD model. The internal device, soldered interconnection, and copper resistances remain identical for all three cases because the device geometry and PCB layout are unchanged. Only the dielectric thermal resistance varies according to the thermal conductivity of FR4, RO4003, or PBO. The calculated resistance components are summarized in Table 2.

The equivalent thermal resistance is obtained by combining the resistance components of the selected heat-transfer path according to Equation (4). The steady-state junction temperature is subsequently calculated using Equation (3), assuming an ambient temperature of 25 °C, a power dissipation of 3.2 W per transistor, and operation without an additional cooling solution. The resulting maximum temperatures, which correspond to the transistor junction temperatures, are presented in Table 3.

The relatively high junction temperatures result from the limited heat-removal capability of the considered structure, particularly because the reduced analytical model retains only the board-directed heat-transfer path. The addition of a heatsink or another dedicated cooling solution would substantially reduce these temperatures. However, its inclusion would require the modeling of additional thermal resistances, thermal-interface contacts, geometrical parameters, and cooling boundary conditions, thereby increasing the complexity of the analytical network. Since the objective of this study is to compare the influence of PCB dielectric-material selection rather than to optimize the complete cooling system, identical simplified thermal conditions are applied to all three configurations.

Under these common conditions, the analytical results predict the same thermal-performance ranking: FR4 produces the highest junction temperature, followed by RO4003, whereas PBO provides the lowest value. Relative to FR4, the calculated junction-temperature reduction is approximately 15.1 °C with RO4003 and 30.8 °C with PBO. These differences result primarily from the lower thermal resistance of the dielectric layer, since the remaining resistance components are maintained constant. The reported temperatures should therefore be interpreted as comparative thermal indicators rather than as recommended continuous operating temperatures.

The implication for heatsink sizing can be expressed through the allowable total thermal resistance. For a prescribed junction-temperature limit $T_{j\;\lim}$, the maximum permissible thermal resistance is determined from $\left(T_{j\;\lim}\;-\;T_{a}\right)\;/\;P_{\mathrm{loss}}$. When the PCB dielectric provides a lower board-directed thermal resistance, a larger portion of this allowable thermal-resistance budget can be assigned to the external cooling system. The required heatsink may therefore have a higher thermal resistance while maintaining the same junction-temperature target. For comparable heatsink technology and ambient conditions, this generally corresponds to a reduced heat-transfer area and potentially lower heatsink volume and mass. However, the exact dimensional reduction depends on the heatsink geometry, airflow, orientation, thermal-interface material, and mounting conditions and is not quantified in the present comparative study.

### 4.2. Numerical Modeling

The numerical modeling process will be divided into two parts. First, Ansys Icepak will be used to perform thermal simulations using the finite element method (FEM). Second, the transistor power loss evolution will be investigated by including parasitic elements extracted using Ansys Q3D regarding each dielectric material in an LTSpice electrical model.

Recent device-level studies reported in the literature further emphasize the need to combine electrical, thermal, and reliability considerations when evaluating GaN technologies. Reference [41] demonstrated that dielectric engineering in enhancement-mode GaN HEMTs can modify gate leakage, transconductance, and gate-related capacitances, showing that dielectric selection may influence both static and high-frequency device behavior. Reference [42] further showed that the intrinsic and extrinsic capacitances and resistances of GaN HEMTs exhibit nonlinear variations with the applied bias conditions, demonstrating the operating-point dependence of the device’s parasitic parameters. From a reliability perspective, [43] highlighted that transient charge accumulation and capacitively coupled paths can generate damaging overvoltages in p-GaN HEMTs. In addition, [44] demonstrated that structural optimization of GaN power devices can redistribute the internal electric field and improve the tradeoff between blocking capability and conduction losses. A recent work [45] also demonstrated the importance of accurate junction-temperature prediction by developing a data-driven model that relates solder-layer void characteristics to the thermal behavior of IGBT modules. Although that study focuses on packaging defects rather than PCB dielectric materials, it reinforces the need to account for material and structural parameters when predicting semiconductor junction temperature. Although these studies concern device-level structures rather than PCB dielectric materials, they collectively support the coupled electrothermal approach adopted in this work, in which material selection is evaluated not only through heat transfer but also through its effects on parasitic capacitances, switching behavior, and device reliability.

#### 4.2.1. Thermal Simulation

The three-dimensional thermal model includes the two GaN devices, soldered interconnections, power-loop copper conductors, a PCB dielectric layer, and external thermal boundaries. The power-loop copper regions are modeled explicitly because they provide the principal heat-spreading paths from the devices toward the PCB.

A constant heat-generation rate of 3.2 W is assigned to the active region of each transistor. The same power dissipation, geometry, dielectric thickness, ambient condition, and external boundary conditions are applied to the FR4, RO4003, and PBO cases. Consequently, the differences observed between the simulations are attributed primarily to the thermal properties of the dielectric materials.

**Figure 12:**
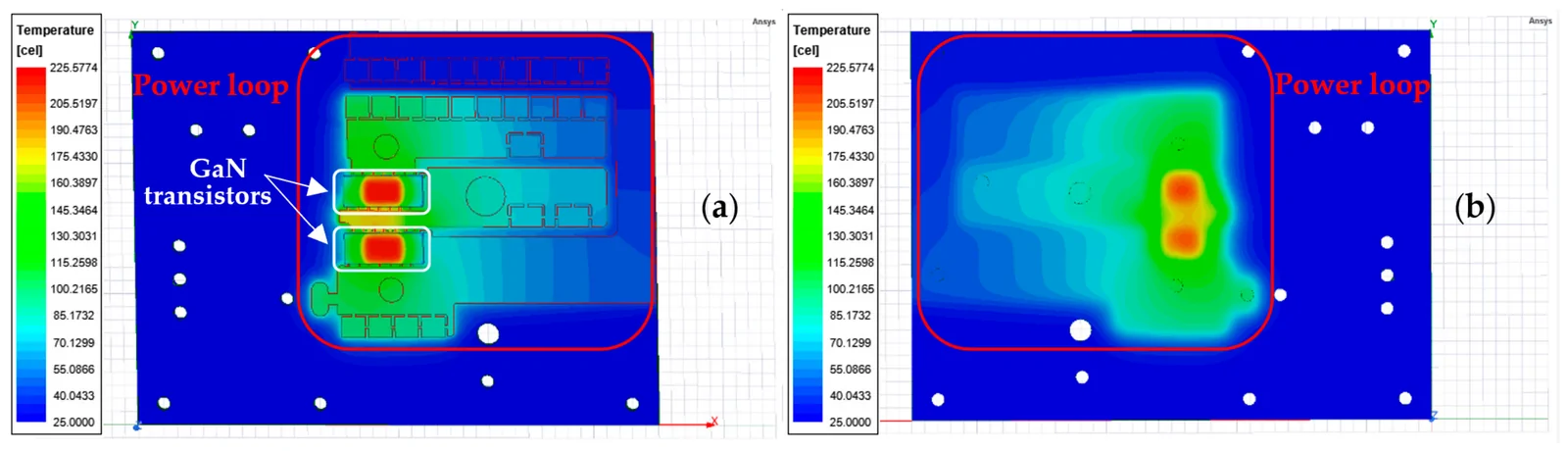
Steady-state temperature distribution obtained with the FR4 dielectric: (**a**) top view and (**b**) bottom view.

**Figure 13:**
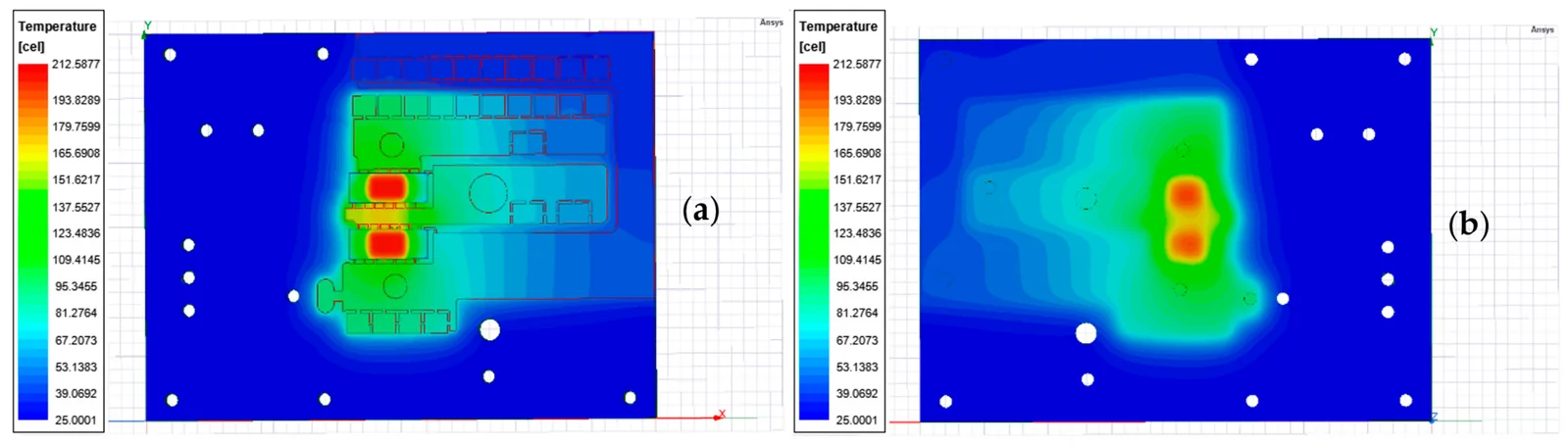
Steady-state temperature distribution obtained with the RO4003 hydrocarbon–ceramic laminate: (**a**) top view and (**b**) bottom view.

**Figure 14:**
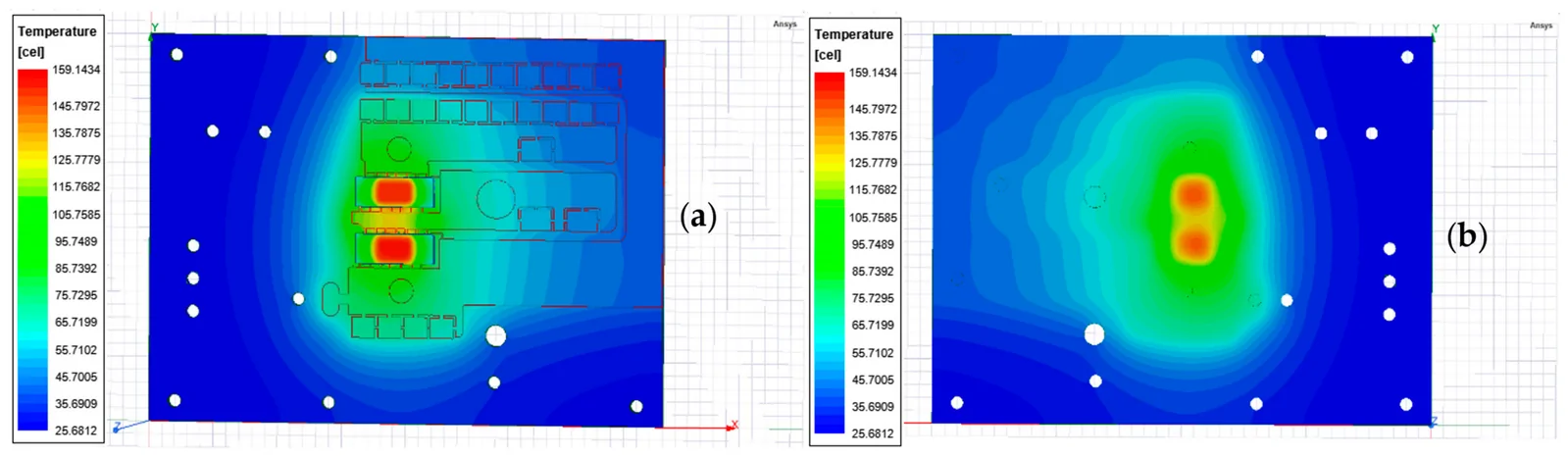
Steady-state temperature distribution obtained with the PBO dielectric: (**a**) top view and (**b**) bottom view.

Figure 12, Figure 13 and Figure 14 present the simulated steady-state temperature distributions for the FR4, RO4003, and PBO cases, respectively. The top views illustrate heat spreading through the power-loop copper regions, whereas the bottom views show the temperature distribution transmitted through the PCB dielectric toward the lower board surface.

The FR4 configuration produces the highest calculated junction temperature, reaching 225 °C, as shown in Figure 12. Replacing FR4 with RO4003 decreases the maximum junction temperature to 212 °C, corresponding to a reduction of 13 °C. The PBO configuration provides the lowest calculated junction temperature, reaching 159 °C, as shown in Figure 14. Compared with FR4, this represents a reduction of 66 °C.

The temperature maps also show that heat is concentrated around the two GaN devices before spreading laterally through the copper conductors and vertically through the PCB structure. The lower temperature obtained with PBO is consistent with its lower dielectric thermal resistance under the identical geometrical and power-dissipation conditions imposed in the simulations.

The FEM results preserve the material ranking predicted by the analytical model: FR4 produces the highest junction temperature, followed by RO4003, whereas PBO provides the lowest temperature. However, the analytical and FEM models do not produce identical absolute values because they represent heat transfer at different levels of detail. The analytical approach uses a reduced one-dimensional resistance network, whereas the FEM model accounts for three-dimensional heat spreading, local temperature gradients, detailed geometry, and numerical boundary conditions.

The calculated junction temperatures are used primarily as comparative thermal-stress indicators. Their high absolute values demonstrate that the selected power-dissipation and cooling conditions represent a severe thermal operating case. They should not be interpreted as recommended continuous operating temperatures. Nevertheless, because identical conditions are imposed for all three materials, the comparison clearly demonstrates the influence of dielectric thermal properties on the board-directed heat-transfer path.

**Figure 15:**
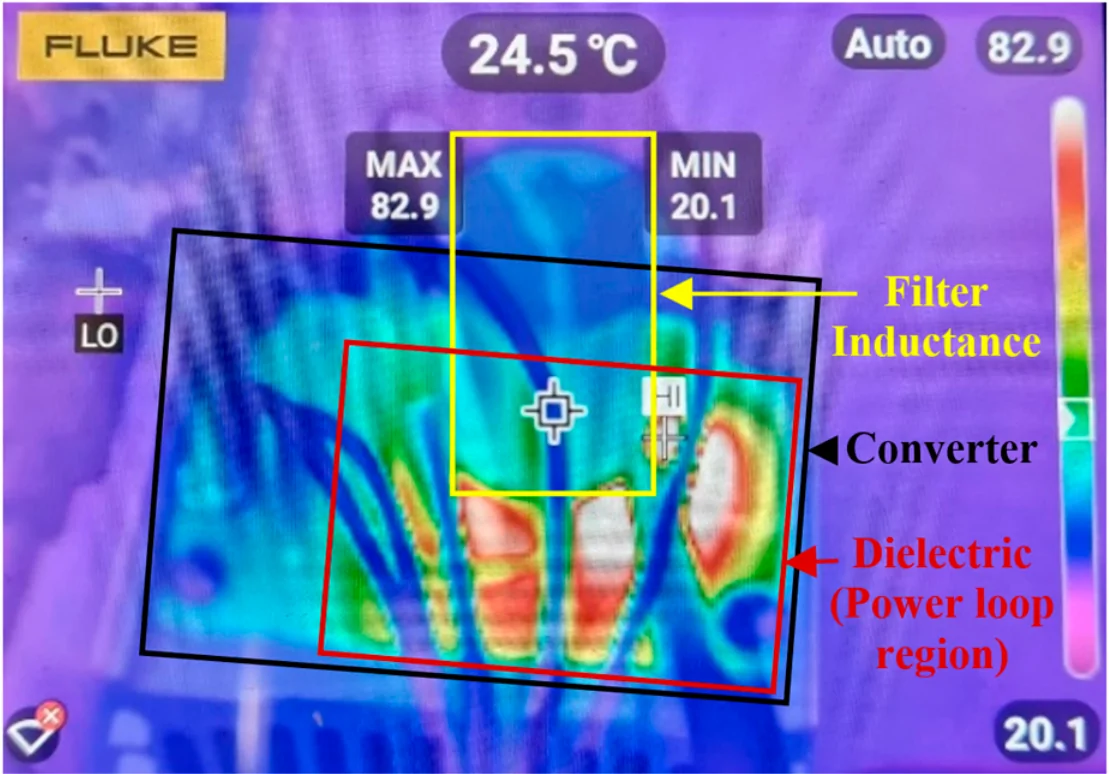
Infrared thermal image of the FR4-based converter operating at an output current of 3 A with forced-air cooling provided by a heatsink and fan.

To provide experimental support for the board-directed heat-transfer mechanism observed in the numerical results, infrared thermography was performed on the manufactured FR4-based converter. The converter was operated at an output current of 3 A while a heatsink and fan provided forced-air cooling to the power stage. As shown in Figure 15, the PCB surface beneath and around the GaN devices reached a maximum measured temperature of 82.9 °C. Although this measurement does not provide the transistor junction temperature, it confirms that a substantial portion of the generated heat is transferred toward the PCB even in the presence of an additional cooling solution. This observation is consistent with the heat-flow path represented in the analytical model and with the temperature distribution predicted by the FEM simulations.

The physical consistency of the steady-state results was also assessed using published thermal information for the EPC2206 and comparable chip-scale GaN devices. The EPC2206 manufacturer documentation identifies simultaneous heat-transfer paths through the PCB-facing interconnections and the device’s top surface and recommends dual-sided cooling for high-power-density operation [46]. This thermal configuration is consistent with the bidirectional heat-flow paths represented in the present numerical model and with the experimentally observed heating of the PCB beneath the devices. Published thermal studies of GaN HEMTs have likewise reported agreement between measured temperature distributions and numerical predictions when the device geometry, heat-source region, material properties, and cooling boundaries are represented appropriately [47]. Because the published configurations differ from the present converter in PCB geometry, mounting, and cooling conditions, their absolute thermal-resistance and temperature values cannot be compared directly. Nevertheless, the reported heat-flow mechanisms and temperature trends support the physical plausibility of the present steady-state simulations.

The present investigation is restricted to steady-state thermal behavior. Accordingly, it predicts the equilibrium junction temperature reached under constant power dissipation but does not describe the heating and cooling trajectories or the thermal time constants of the converter. A transient analysis would require the complete thermal-capacitance network, time-dependent power profiles, and appropriate initial conditions. The present results should therefore be interpreted as a comparison of the final thermal equilibrium obtained with the three dielectric materials rather than as a prediction of their temporal responses.

The present numerical model focuses on the power stage and does not explicitly include the gate-driver circuit. Consequently, the reduction in GaN junction temperature obtained with a higher-thermal-conductivity dielectric cannot be directly extended to the gate driver. Under practical operating conditions, enhanced lateral heat spreading through the PCB may transfer part of the heat generated by the GaN devices toward nearby components. If the gate driver is located close to the power stage, the resulting temperature rise may modify temperature-dependent electrical parameters, such as the output-stage resistance and propagation characteristics, and may consequently affect the gate-current profile and switching behavior. Conversely, improved vertical heat conduction through the PCB may reduce the average board temperature and limit this thermal interaction. A quantitative assessment would require the gate-driver package, dissipated power, temperature-dependent properties, and exact placement to be incorporated into the three-dimensional model.

Although PBO yields the strongest thermal improvement among the investigated materials, the present results alone do not directly determine the required heatsink size or cost. A quantitative cooling-system reduction would require an additional design calculation based on the permissible junction temperature, ambient temperature, device losses, and required heatsink-to-ambient thermal resistance.

#### 4.2.2. Parasitic Capacitance and Power-Loss Evaluation

The influence of the dielectric material on the electrical behavior of the converter was evaluated by extracting the layout-associated parasitic capacitances using Ansys Q3D. The converter geometry and conductor arrangement were kept identical in all cases; only the dielectric material properties were modified. Table 4 presents the extracted capacitances associated with the high-side region, switching-node region, and low-side region.

$C_{\mathrm{HS}\;}$, $C_{\mathrm{SN}}$, and $C_{\mathrm{LS}}$ denote the extracted capacitances associated with the high-side, switching-node, and low-side copper regions, respectively.

The extracted results show that PBO produces the lowest capacitance values for all three power-loop regions. Relative to FR4, the PBO configuration decreases $C_{\mathrm{HS}\;}$, $C_{\mathrm{SN}}$, and $C_{\mathrm{LS}}$ by approximately 35.3%, 37.7%, and 30.1%, respectively. RO4003 provides an intermediate reduction, consistent with its relative permittivity lying between those of FR4 and PBO.

Because the conductor geometry and separation distances are identical in the three models, these differences are primarily attributed to the change in dielectric permittivity and the resulting electric-field distribution. Lower layout-associated capacitance reduces the capacitive loading of the switching node and the energy exchanged during each voltage transition. It may therefore influence the switching transition duration, displacement currents, ringing, and switching losses.

However, the extracted capacitance values alone do not demonstrate compliance with electromagnetic compatibility limits. A quantitative EMC assessment would require conducted- or radiated-emission simulations or measurements. The present analysis is consequently restricted to evaluating the influence of dielectric selection on parasitic capacitance and switching behavior.

At the first order, the energy associated with charging a parasitic capacitance through a voltage excursion ΔV is proportional to $\frac{1}{2}C_{p}{\left(\Delta V\right)}^{2}$. For repeated switching at frequency $f_{\mathrm{sw}}$, the corresponding power contribution scales with $C_{p}{\left(\Delta V\right)}^{2}f_{\mathrm{sw}}$. Therefore, the electrical influence of dielectric-material selection is expected to become more pronounced as the switching voltage and frequency increase.

The total losses of a GaN half-bridge include conduction losses, switching-transition losses, output-capacitance-related losses, and reverse-conduction losses during the dead time. Because GaN HEMTs do not contain an intrinsic body diode, reverse current is conducted through the device channel under negative drain-to-source voltage. The associated voltage drop can produce significant losses when the dead time is unnecessarily long [48,49,50]. Therefore, identical dead time, gate-drive, load, and switching-frequency conditions are imposed for all dielectric-material cases.

The switching-node transition time is governed by several interacting parameters, including the gate resistance, driver current capability, load current, nonlinear transistor capacitances, power-loop parasitic elements, and layout-associated capacitances. In the present comparative study, the transistor model, gate-driver parameters, load condition, parasitic inductances, and conductor geometry are kept unchanged. Only the capacitances extracted for the FR4, RO4003, and PBO dielectric configurations are modified in the LTspice model.

Consequently, the differences observed in the simulated falling time are interpreted as the incremental influence of the PCB dielectric material rather than as a complete representation of all mechanisms governing the switching transition. Parasitic inductances are not varied because the conductor geometry is identical for all three cases. Their exclusion from the comparative calculation limits the prediction of the absolute ringing behavior but does not prevent evaluation of the relative capacitance-induced differences between the dielectric materials.

**Figure 16:**
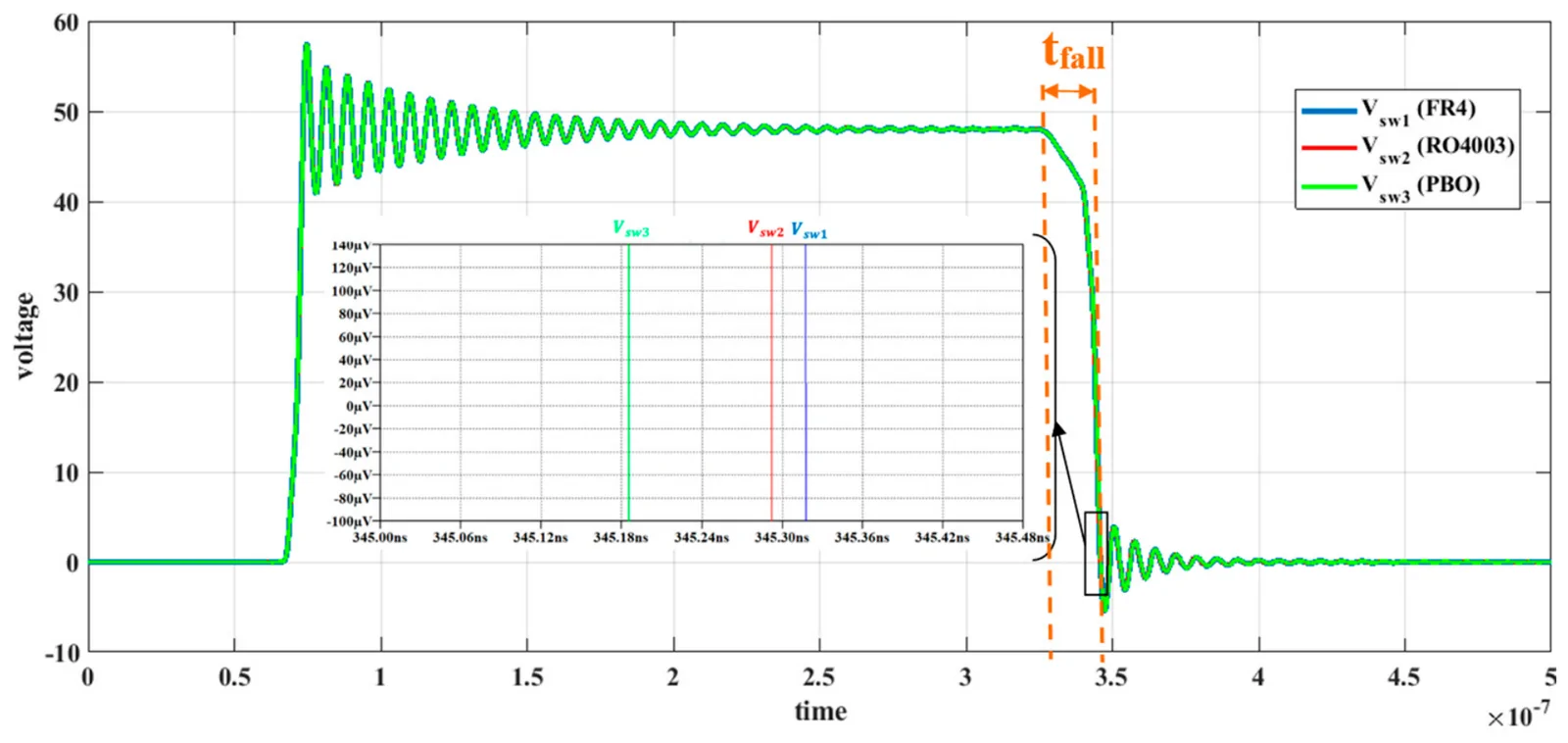
Simulated switching-node falling transitions obtained using the parasitic capacitances extracted for FR4, RO4003, and PBO.

LTspice simulations were performed to quantify the influence of the extracted capacitances on the switching-node falling time, $t_{\mathrm{fall}}$, for the three dielectric configurations, as presented in Figure 16. The blue, red, and green curves represent, respectively, the Vsw signal using FR4, RO4003, and PBO dielectric materials.

The simulated falling times are 13.318 ns, 13.292 ns, and 13.185 ns for the FR4, RO4003, and PBO configurations, respectively. Relative to FR4, RO4003 reduces the falling time by approximately 0.026 ns, whereas PBO provides a reduction of approximately 0.133 ns, corresponding to about 1.0%. Although the absolute differences are small, the consistent trend indicates that the reduction in layout-associated capacitance slightly accelerates the switching-node transition under otherwise identical simulation conditions.

The present loss calculation is intentionally formulated as a first-order comparative model rather than as a complete prediction of all device-loss mechanisms. Its purpose is to isolate the incremental influence of the dielectric-dependent layout capacitances on the transistor losses while keeping the device model, gate-drive parameters, load current, switching frequency, duty cycle, dead time, and remaining parasitic elements identical for the three configurations. Under these controlled conditions, the conduction contribution remains unchanged, whereas the variation in the switching-transition loss is governed primarily by the simulated rise and fall times. The total transistor loss is therefore approximated as the sum of the conduction and voltage–current overlap switching-loss contributions given by Equations (5)–(7) [40]:

(5) $$P_{Loss\_GaN}\;=\;P_{Switching}\;+\;P_{Cond}$$

where

- $P_{cond}$ is the power loss of the GaN device under the conduction state.

(6) $$P_{cond}=Rds_{\left(on\right)}\left[Ω\right]\;*\;I^{2}\left[A\right]\;*\;D$$

- $P_{Switching}$ is the power loss of the GaN device under the switching state.

(7) $$P_{Switching}=P_{On}+P_{Off}=\frac{\left(V_{max}\;*\;I_{max}\right)}{2}\left(t_{rise}+t_{fall\;}\left[s\right]\right)f$$

- $P_{On}$ is the power loss during the rising time “trise”, as shown in Figure 2.
- $P_{Off}$ is the power loss during the falling time “tfall”, as indicated in Figure 2.

Here,

$V_{max}\;=\;48\;V;\;I_{max}\;=\;10\;A;\;$f = 1 MHz and D: Duty cycle

This reduced formulation does not explicitly separate nonlinear output-capacitance losses, reverse-conduction losses during dead time, gate-drive losses, or temperature-dependent variations in RDS (on). These contributions are either embedded in the transistor simulation model or remain effectively identical among the three dielectric cases because the corresponding electrical and operating parameters are unchanged. Consequently, the model is suitable for comparing the relative loss variation caused by dielectric-material selection, although it should not be interpreted as a complete absolute loss model of the converter.

The resulting transistor losses are summarized in Table 5. Because all electrical parameters other than the extracted layout-associated capacitances are kept constant, the reported differences represent only the incremental influence of dielectric-material selection on the simulated switching transition.

To reduce overshoots, the rising time “$t_{rise\;}$” can be adjusted by varying the resistance values at the gate driver output. However, increasing the “$t_{rise\;}$” will subsequently increase the switching losses.

The calculated loss decreases from 3.118 W for FR4 to 3.102 W for PBO, corresponding to a reduction of 16 mW, or approximately 0.51%. The RO4003 case produces an intermediate reduction of 4 mW. These results show that the direct electrical-loss improvement remains modest for the investigated converter and operating conditions.

Under steady-state conditions, the thermal effect of the calculated loss reduction is governed by the corresponding thermal resistance and does not accumulate continuously with operating time. Therefore, the substantial junction-temperature reduction observed in the FEM analysis is primarily attributed to the lower thermal resistance of the dielectric material rather than to the 16 mW reduction in transistor loss.

It should also be noted that the FEM simulations were performed using the same assigned heat-generation rate for all three materials. Consequently, the thermal and electrical analyses presently constitute a one-way comparative study. A fully coupled electrothermal analysis would require the Icepak model to be rerun using the specific calculated power loss obtained for each dielectric configuration.

The electrical results presented in this study are specific to the investigated converter geometry and operating conditions. Layout-associated capacitances depend not only on dielectric permittivity but also on conductor area, separation distance, copper thickness, layer arrangement, and the surrounding electrical environment. Consequently, the reported capacitance and power-loss values should not be extrapolated directly to converters with different layouts or power ratings.

For the investigated one-sided structure, dielectric-material selection primarily influences the thermal resistance of the board-directed heat-transfer path, while its effect on the calculated electrical losses remains comparatively small. Nevertheless, the electrical contribution may become more significant at higher switching frequencies or larger voltage excursions, or in multilayer structures exhibiting stronger capacitive coupling. These conditions should be investigated using the same coupled extraction and simulation methodology.

Future work will therefore focus on transient electrothermal modeling and experimental validation of the predicted temperature and switching-waveform differences, evaluation of additional operating points, and implementation of a fully coupled electrothermal model in which the material-dependent electrical losses are introduced directly into the thermal simulation. The influence of dielectric thickness, multilayer PCB structures, temperature-dependent material properties, and alternative cooling boundary conditions will also be examined.

## 5. Conclusions

This study investigated the influence of PCB dielectric-material selection on the thermal and electrical behavior of a high-power-density GaN half-bridge converter. FR4, RO4003, and PBO were compared using a reduced analytical thermal-resistance network, three-dimensional FEM simulations in Ansys Icepak, parasitic-capacitance extraction in Ansys Q3D, and switching simulations in LTspice.

Both the analytical and FEM models produced the same thermal-performance ranking. FR4 resulted in the highest junction temperature, RO4003 provided an intermediate improvement, and PBO produced the lowest temperature. In the FEM analysis, replacing FR4 with PBO reduced the calculated maximum junction temperature from 225 °C to 159 °C under identical geometry, power dissipation, and boundary conditions. This improvement was primarily attributed to the lower thermal resistance of the PBO dielectric layer.

The electrical analysis also showed that PBO produced the lowest layout-associated parasitic capacitances. Relative to FR4, the extracted capacitances were reduced by approximately 30–38%, leading to a slight reduction in the simulated switching-node falling time and a calculated transistor-loss decrease from 3.118 W to 3.102 W. Therefore, the direct electrical-loss benefit remained modest under the investigated operating conditions, whereas the principal advantage of the dielectric modification was thermal. This analysis was intended to quantify the contribution of dielectric-dependent parasitic capacitances to switching losses and heat generation, rather than assessing the EMI behavior.

The results demonstrate that PCB dielectric selection should be treated as a coupled electrothermal design parameter in high-frequency GaN converters. Reducing the thermal resistance of the board-directed heat-transfer path increases the allowable thermal resistance of the external cooling system for a prescribed junction-temperature limit. Under equivalent cooling technology and ambient conditions, this may permit the use of a heatsink with a smaller heat-transfer area, volume, and mass. However, the exact dimensional benefit depends on the heatsink geometry, airflow, orientation, mounting conditions, and thermal-interface properties and was not quantified in this study. Future work will focus on experimental validation, additional operating points, temperature-dependent material properties, multilayer PCB structures, and fully coupled electrothermal simulations using the dielectric-dependent power losses as thermal inputs.

## Figures and Tables

**Table 1:** The relative permittivity for each dielectric material.

Dielectric Material	FR4	RO4003	PBO
Relative permittivity (F/m)	4.5	3.38	3

**Table 2:** Thermal resistances for each material in [°C/W].

$R_{\theta JC}$	$R_{\theta \_Solder}$	$R_{\theta \_Pads}$	$R_{\theta \_Copper}$	$R_{\theta \_Dielectric}$			$R_{\theta_{TB}}$
				FR4	RO4003	PBO	
0.376	0.335	0.178	0.275	10.38	5.67	0.725	47.730

**Table 3:** Analytical steady-state junction temperatures for the three dielectric materials.

Dielectric Material	Tj [°C]
FR4	214.67
RO4003	199.60
PBO	183.87

**Table 4:** Comparative table of the power loop’s parasitic capacitances for each material.

Material	C_HS [pF]	C_SN [pF]	C_LS [pF]
1	3.29	4.27	4.19
2	2.59	3.30	3.43
3	2.13	2.66	2.93

**Table 5:** Calculated EPC2206 power losses using the parasitic capacitances associated with each dielectric configuration.

Dielectric Material	Power Losses (W)
FR4	3.118
RO4003	3.114
PBO	3.102

## Data Availability

The original contributions presented in this study are included in the article. Further inquiries can be directed to the corresponding author.

## Abbreviations

The following abbreviations are used in this manuscript:

FEM	Finite Elements Method
FR4	Flame Retardant 4
RO4003	Hydrocarbon ceramic laminate material RO4000 series (4003)
PBO	Polybenzoxazole
PCB	Printed Circuit Board

## References

[1] Jones E.A., Wang F.F., Costinett D. (2016). Review of commercial GaN power devices and GaN-based converter design challenges. IEEE J. Emerg. Sel. Top. Power Electron..

[2] Zhang W., Huang X., Liu Z., Lee F.C., She S., Du W., Li Q. (2015). A new package of high-voltage cascode gallium nitride device for megahertz operation. IEEE Trans. Power Electron..

[3] Brothers J.A., Beechner T. (2019). GaN module design recommendations based on the analysis of a commercial 3-phase GaN module. 2019 IEEE Energy Conversion Congress and Exposition (ECCE).

[4] Passmore B., Storkov S., McGee B., Stabach J., Falling G., Curbow A., Killeen P., Flint T., Simco D., Shaw R. (2015). A 650 V/150 A enhancement mode GaN-based half-bridge power module for high frequency power conversion systems. 2015 IEEE Energy Conversion Congress and Exposition (ECCE).

[5] Kou L., Lu J. (2020). Applying GaN HEMTs in conventional housing-type power modules. 2020 IEEE Energy Conversion Congress and Exposition (ECCE).

[6] Qi Z., Pei Y., Wang L., Yang Q., Wang K. (2021). A highly integrated PCB embedded GaN full-bridge module with ultralow parasitic inductance. IEEE Trans. Power Electron..

[7] Savulak S., Guo B., Krishnamurthy S. (2018). Three-phase inverter employing PCB embedded GaN FETs. 2018 IEEE Applied Power Electronics Conference and Exposition (APEC).

[8] Moench S., Reiner R., Waltereit P., Benkhelifa F., Huckelheim J., Meder D., Zink M., Kaden T., Noll S., Mansfeld S. (2020). PCB-embedded GaN-on-Si half-bridge and driver ICs with on-package gate and DC-link capacitors. IEEE Trans. Power Electron..

[9] Emon A.I., Carlton H., Harris J., Krone A., Hassan M.U., Mirza A.B., Hossain M., Rashid A.U., Chen Y., Luo F. (2022). Design and optimization of gate driver integrated multichip 3-D GaN power module. IEEE Trans. Transp. Electrif..

[10] Lu J.L., Chen D., Yushyna L. (2017). A high power-density and high efficiency insulated metal substrate based GaN HEMT power module. 2017 IEEE Energy Conversion Congress and Exposition (ECCE).

[11] Jørgensen A.B., Bęczkowski S., Uhrenfeldt C., Petersen N.H., Jørgensen S., Munk-Nielsen S. (2018). A fast-switching integrated full-bridge power module based on GaN eHEMT devices. IEEE Trans. Power Electron..

[12] Yan Y., Zhu L., Walden J., Liang Z., Bai H., Kao M.H. (2021). Packaging a top-cooled 650 V/150 A GaN power modules with insulated thermal pads and gate-drive circuit. 2021 IEEE Applied Power Electronics Conference and Exposition (APEC).

[13] Emon A.I., Carlton H., Harris J., Krone A., Hassan M., Mirza A., Yuan Z., Huitink D., Luo F. (2021). A 650V/60A gate driver integrated wire-bondless multichip GaN module. 2021 IEEE 12th International Symposium on Power Electronics for Distributed Generation Systems (PEDG).

[14] Tian X., Jia N., DeVoto D., Paret P., Bai H., Tolbert L.M., Cui H. (2023). PCB-on-DBC GaN Power Module Design with High-Density Integration and Double-Sided Cooling. IEEE Trans. Power Electron..

[15] Lumbreras D., Vilella M., Zaragoza J., Berbel N., Jordà J., Collado A. (2021). Effect of the heat dissipation system on hard-switching GaN-based power converters for energy conversion. Energies.

[16] Sodan V., Stoffels S., Oprins H., Decoutere S., Altmann F., Baelmans M., De Wolf I. (2018). Fast and distributed thermal model for thermal modeling of GaN power devices. IEEE Trans. Compon. Packag. Manuf. Technol..

[17] Mitterhuber L., Hammer R., Dengg T., Spitaler J. (2020). Thermal characterization and modelling of AlGaN-GaN multilayer structures for HEMT applications. Energies.

[18] GaN Systems (2020). Thermal Design for GaNPX Packaged Devices.

[19] Plexim GmbH (2019). PLECS User Manual Version 4.4.

[20] Górecki K., Zarębski J., Górecki P., Ptak P. (2019). Compact thermal models of semiconductor devices—A review. Int. J. Electron. Telecommun..

[21] Posobkiewicz K., Górecki K., Zarębski J. (2021). Influence of selected factors on thermal parameters of the components of forced cooling systems of electronic devices. Electronics.

[22] Janicki M., Ptak P., Torzewicz T., Górecki K. (2020). Compact thermal modeling of modules containing multiple power LEDs. Energies.

[23] Bahman A.S., Ma K., Ghimire P., Iannuzzo F., Blaabjerg F. (2016). A 3D lumped thermal network model for long-term load profiles analysis in high-power IGBT modules. IEEE J. Emerg. Sel. Top. Power Electron..

[24] Cheng T.-H., Hopkins D., Beczkowski S., Uhrenfeldt C., Munk-Nielsen S. (2023). A monolithic GaN driver with a deadtime generator (DTG) for high-temperature GaN power converters. IET Power Electron..

[25] Chvála A., Marek J., Príbytný P., Šatka A., Stoffels S., Posthuma N., Decoutere S., Donoval D. (2017). Analysis of multifinger power HEMTs supported by effective 3-D device electrothermal simulation. Microelectron. Reliab..

[26] Cova P., Delmonte N., Santoro D. (2019). Power GaN FET boards thermal and electromagnetic optimization by FE modeling. Microelectron. Reliab..

[27] Patel A., Munk-Nielsen S. (2022). A survey of conductive and radiated EMI reduction techniques in GaN-based converters. IET Power Electron..

[28] Zhang S., Laboure E., Labrousse D., Lefebvre S. (2017). Thermal management for GaN power devices mounted on PCB substrates. 2017 IEEE International Workshop On Integrated Power Packaging (IWIPP).

[29] Kalantarian A., Jin M., Merkelbach J., Yusufali J. (2017). Evaluation of high thermal conductivity dielectric on the temperature of PCB components. 2017 16th IEEE Intersociety Conference on Thermal and Thermomechanical Phenomena in Electronic Systems (ITherm).

[30] Yu C., Buttay C., Laboure E. (2016). Thermal management and electromagnetic analysis for GaN devices packaging on DBC substrate. IEEE Trans. Power Electron..

[31] Joo D., Kim H.B., Lee B.K., Kim J.S. (2019). Hardware implementation of GaN-HEMT based ZVS DC–DC converter considering PCB layout. J. Electr. Eng. Technol..

[32] EPC (2021). EPC2206 Datasheet. https://epc-co.com/epc/Portals/0/epc/documents/datasheets/EPC2206_datasheet.pdf.

[33] Fang L., Rosset Y., Sarrazin B., Lefranc P., Rio M. (2025). Parametric LCA model for power electronic ecodesign process: Addressing MOSFET-Si and HEMT-GaN technological issues. IET Power Electron..

[34] Hou R., Lu J., Chen D. (2018). Parasitic capacitance Eqoss loss mechanism, calculation, and measurement in hard-switching for GaN HEMTs. 2018 IEEE Applied Power Electronics Conference and Exposition (APEC).

[35] Xu J., Qiu Y., Chen D., Lu J., Hou R., Di Maso P. (2017). An Experimental Comparison of GaN E-HEMTs Versus SiC MOSFETs Over Different Operating Temperatures.

[36] Sheng W.W., Colino R.P. (2004). Power Electronic Modules: Design and Manufacture.

[37] Randoll R., Wondrak W., Schletz A. (2014). Dielectric strength and thermal performance of PCB-embedded power electronics. Microelectron. Reliab..

[38] Li L., Jiang Z., Jiang Y. (2023). A SEPIC flyback DC-DC converter with GaN switching device. J. Electr. Eng. Technol..

[39] Belguith M., Eloued S., Kadi M., Slama J.B.H. One Sided GaN-Based Buck Converter Layout Optimization by Minimizing Power Loop Parasitics and Considering Heat Dissipation Efficiency. Proceedings of the 2023 IEEE International Conference on Artificial Intelligence & Green Energy (ICAIGE).

[40] Besserour M.A., Eloued S., Slama J.B.H., Fouquet F., Kadi M. Thermal Modelling for an Electrothermal Model of GaN Devices. Proceedings of the 2021 12th International Renewable Energy Congress (IREC).

[41] Li Y., Huang Y., Li J., Sun H., Guo Z. (2025). Effect of Dual Al_2_O_3_ MIS Gate Structure on DC and RF Characteristics of Enhancement-Mode GaN HEMT. Micromachines.

[42] Alim M.A., Gaquiere C. (2025). Impact of Multi-Bias on the Performance of 150 nm GaN HEMT for High-Frequency Applications. Micromachines.

[43] Shi Y., Chen Y., He L., Chen X., Chen Y., Lu G. (2025). The ESD Robustness and Protection Technology of P-GaN HEMT. Micromachines.

[44] Yu Y.S., Yoon S., Oh J.H. (2026). Design and Simulation of a High-Performance GaN Vertical Merged P-i-N/Schottky (MPS) Diode with Multi-Drift-Layer and Field-Plate Termination. Micromachines.

[45] Wei Y., Yang X., Wu P., Liu G. (2026). A Data-Driven Approach to Predict the Impact of Void Characteristics on IGBT Junction Temperature. IEEE Trans. Electron Devices.

[46] Efficient Power Conversion Corporation (2026). EPC2206—Automotive 80 V (D–S) Enhancement-Mode Power Transistor. EPC2206 Datasheet. EPC Official Product Documentation. https://assets.sourcengine.com/datasheets/3993bd17-adfd-4718-8c5b-bacd4d60a077.pdf.

[47] Das J., Oprins H., Ji H., Sarua A., Ruythooren W., Derluyn J., Kuball M., Germain M., Borghs G. (2006). A temperature analysis of high-power AlGaN/GaN HEMTs. Solid-State Electron..

[48] Hou R., Shen Y., Zhao H., Hu H., Lu J., Long T. (2020). Power loss characterization and modeling for GaN-based hard-switching half-bridges considering dynamic on-state resistance. IEEE Trans. Transp. Electrif..

[49] GaN Systems (2018). GS66508P Bottom-Side Cooled 650 V E-Mode GaN Transistor Datasheet.

[50] GaN Systems (2020). GS66508T Top-Side Cooled 650 V E-Mode GaN Transistor Preliminary Datasheet.

